# Pharmacist-Led Education Intervention for Adults With Allergic Rhinitis: A Randomized Clinical Trial

**DOI:** 10.1001/jamanetworkopen.2025.17160

**Published:** 2025-07-16

**Authors:** Chii-Chii Chew, Xin-Jie Lim, Pathma Letchumanan, Philip Rajan, Chee Ping Chong

**Affiliations:** 1 Clinical Research Centre, Hospital Raja Permaisuri Bainun, Institute for Clinical Research, National Institute of Health, Ministry of Health Malaysia, Ipoh, Perak, Malaysia; 2 Discipline of Clinical Pharmacy, School of Pharmaceutical Sciences, Universiti Sains Malaysia, Minden, Penang, Malaysia; 3 Clinical Research Centre, Hospital Raja Permaisuri Bainun, Ministry of Health, Ipoh, Perak, Malaysia; 4 Department of Otorhinolaryngology, Hospital Raja Permaisuri Bainun, Ministry of Health, Ipoh, Perak, Malaysia

## Abstract

**Importance:**

Allergic rhinitis (AR) affects 10% to 30% of the adult population globally. The current framework of pharmaceutical care for managing AR focuses on community pharmacies, with limited structured pharmaceutical care in public health care settings.

**Objective:**

To evaluate the effectiveness of a pharmacist-led education intervention (AR-PRISE) in a public health care institution compared with standard care for managing AR among adults.

**Design, Setting, and Participants:**

This randomized clinical trial used a 2-arm, parallel-group, open-label design in the otorhinolaryngology clinic of a government-funded tertiary referral hospital in northern Malaysia. Enrollment occurred from June 1, 2023, to February 15, 2024, with follow-up completed August 6, 2024. The trial included Malaysian patients aged 18 to 80 years who had received a diagnosis of AR and were able to comprehend English or Malay. Of the 209 screened patients, 154 were recruited and randomly assigned into intervention and control groups, with 149 completing the study.

**Interventions:**

The AR-PRISE intervention involved an 8-minute educational video on AR and structured pharmacist counseling.

**Main Outcomes and Measures:**

The primary outcomes were between-group differences at day 180 in knowledge level, symptom control (Total Nasal Symptom Score [TNSS] for the past 12 hours and past 2 weeks), medication adherence (number of days of intranasal corticosteroid use), and quality of life (assessed by the European Quality of Life 5-Dimension 5-Level Instrument and European Quality of Life Visual Analog Scale). Assessments were conducted at baseline and days 60 (±7), 120 (±7), and 180 (±7). All analyses were performed on an intention-to-treat basis.

**Results:**

Among the 154 participants, the mean (SD) age was 46.5 (17.0) years, and 97 (63.0%) were women; 77 participants were randomized to each group. In the intention-to-treat analysis, the intervention group showed statistically significant improvement in TNSSs for the past 2 weeks compared with controls (estimate [SE], 0.14 [0.06]; 95% credible interval [CrI], 0.03-0.25). Significant reductions in the TNSS for the past 2 weeks were observed at all time points (day 60: estimate [SE], 0.18 [0.05]; 95% CrI, 0.08-0.28; day 120: estimate [SE], 0.21 [0.05]; 95% CrI, 0.11-0.31; and day 180: estimate [SE], 0.11 [0.05]; 95% CrI, 0.01-0.21). No significant differences were observed for knowledge level, TNSS for the past 12 hours, medication adherence, or quality of life.

**Conclusions and Relevance:**

In this randomized clinical trial, the AR-PRISE intervention significantly improved AR symptom control (TNSS for the past 2 weeks) compared with standard care over 180 days but did not show significant effects on other coprimary outcomes (AR knowledge, TNSS for the past 12 hours, medication adherence, or quality of life). These findings suggest that while pharmacist-led education can enhance symptom management, additional strategies may be needed to address broader aspects of AR care.

**Trial Registration:**

ClinicalTrials.gov Identifier: NCT06027736

## Key Points

**Question:**

Is a pharmacist-led education intervention more effective than standard care for managing allergic rhinitis (AR) in adults?

**Findings:**

In this randomized clinical trial of 154 adults with AR, the pharmacist-led education intervention significantly improved AR symptom control (measured by 2-week Total Nasal Symptom Scores [TNSSs]) compared with standard care over 180 days. However, there were no significant differences between groups in the coprimary outcomes: 12-hour TNSS, AR knowledge level, medication adherence, or quality of life.

**Meaning:**

The pharmacist-led education intervention provided sustained relief of nasal symptoms among patients with AR but did not improve immediate symptoms, knowledge, adherence, or quality of life vs standard care.

## Teaser

This randomized clinical trial evaluates the effectiveness of a pharmacist-led education intervention compared with standard care for managing nasal symptoms of allergic rhinitis among adults, as well as allergic rhinitis knowledge, medication adherence, and quality of life.

## Introduction

Allergic rhinitis (AR) affects 10% to 30% of adults globally, imposing a substantial burden on health, well-being, and the economy.^1^ As a common comorbidity of asthma, AR exacerbates respiratory symptoms and increases health care use through more frequent medical visits.^2^ Allergic rhinitis impairs quality of life (QOL) by reducing work productivity and academic performance while generating considerable health care costs.^3^ Studies demonstrate that pharmacists play a vital role in AR management through patient education, pharmacologic recommendations, and self-management support,^4,5,6,7,8,9,10^ with proven effectiveness in improving symptom control and QOL.^5,11^ However, to date, the role of pharmacists in AR management remains underused.

Pharmacy practices and health care settings vary significantly across countries, particularly in 2-tiered health care systems combining public and private sectors. These systems typically comprise (1) publicly funded government health care and (2) private sector services relying on out-of-pocket payments or private insurance. This distinction creates fundamentally different practice environments for pharmacists.^12^ Although community pharmacies in the private sector have established AR management protocols under Allergic Rhinitis and its Impact on Asthma (ARIA) pharmacy guidelines, government health care institutions lack standardized pharmaceutical care frameworks for AR.^7^

To address this unmet need, we developed the pharmacist-led education intervention (AR-PRISE) model, which integrated audiovisual patient education with structured counseling. The concept and details of the development are described elsewhere.^13,14^ This model provides a structured framework for AR management in public health care services by shifting from physician-centric care to a collaborative approach that leverages pharmacists’ medication expertise. The study evaluated the effectiveness of AR-PRISE compared with standard care for managing adult patients with AR in a public health care institution.

## Methods

### Study Design

This study was a single-center, open-label, parallel-group randomized clinical trial. Enrollment occurred from June 1, 2023, to February 15, 2024, with follow-up completed August 6, 2024. The study received approval from the Medical Research and Ethics Committee of the Ministry of Health of Malaysia. Written informed consent was obtained from each participant. We adhered to the Consolidated Standards of Reporting Trials (CONSORT) reporting guideline, with the trial protocol detailed in Supplement 1 and previously published.^15^

### Study Population

All consecutive adult patients (aged 18-80 years) who received a diagnosis of AR and were attending a tertiary hospital otorhinolaryngology outpatient clinic during the study period were screened for enrollment. Exclusion criteria included (1) concomitant chronic rhinosinusitis, sinusitis, nasal polyposis, nasal septal deviation, sinonasal malignant neoplasm, and terminal illnesses; (2) pregnancy; (3) long COVID; (4) preexisting psychiatric conditions or dementia making the patient unable to answer the questionnaire; and (5) insufficient proficiency in English or Malay.

### Randomization, Blinding, and Allocation

Participants were randomly allocated in a 1:1 ratio to either the intervention or control group. An independent research assistant generated this randomization sequence using a web-based randomization program (Sealed Envelope, version 1.21.0 [Sealed Envelope Ltd]) with permuted block sizes of 4, 6, and 8. The research assistant, who was not involved in patient treatment or recruitment, stored the randomization list in a password-protected file to ensure concealment until assignment. On receiving consent from the participants and conducting the initial assessment, the study personnel contacted the independent research assistant by telephone. The independent research assistant revealed the randomization assignment of the participants to either the intervention or control groups. The study team was blinded to treatment allocation (allocation sequence concealment). Due to the nature of the interventions, blinding was not possible for participants or interventionists, and study staff did not require blinding procedures for outcome assessment because the outcomes were self-reported and therefore reduced assessor bias.

### Intervention

This study implemented the pharmacist-led education intervention (AR-PRISE). On completion of the baseline assessment, participants were required to watch an 8-minute educational video on AR and received structured pharmacist counseling. The video covered the following 10 key elements: (1) background; (2) symptoms; (3) diagnosis; (4) allergic identification and avoidance; (5) intranasal corticosteroid (INCS) use, consisting of general information, priming, administration techniques, cleaning, and addressing concerns; (6) antihistamines; (7) decongestants; (8) alkaline nasal douches; (9) what to do when symptoms flare; and (10) consequences of poor disease control. The pharmacist’s counseling included assessing symptom severity and QOL, setting treatment goals, evaluating INCS administration techniques, addressing treatment concerns, and highlighting the consequences of nonadherence. Patients were informed about the importance of allergen identification and avoidance and were encouraged to periodically review educational materials. In addition, they were made aware of the signs of asthma exacerbation, especially if they had coexisting AR and asthma.

Participants received a diary booklet to document their medication use. The booklet contained a QR code that provided direct access to the educational videos, enabling the intervention group participants to review the material at home, while the control group did not receive the QR code. During follow-up visits on days 60 (±7) and 120 (±7), the pharmacist underscored the importance of adherence to the intervention and offered assistance as required.

### Standard Care

Participants in the control group received standard care for AR management, which consisted of physician consultations at the otorhinolaryngology clinic and routine medication counseling when filling their prescriptions at an outpatient pharmacy. Throughout the study, the control group did not receive any additional interventions or instructions to view educational videos.

### Measures

The study collected sociodemographic information including age, sex, race and ethnicity (extracted from participants’ clinic cards for comparison with national distributions), marital status, educational level, occupation, working hours, and smoking status. Other baseline data included number of pets at home, number of food allergies, number of other allergens, history of nasal surgery, family history of allergy, comorbidities, and medications.

The severity of AR symptoms was measured longitudinally using a visual analog scale (VAS) at baseline and on day 180 (±7), and the ARIA classification was assessed at baseline and days 60 (±7), 120 (±7), and 180 (±7). The VAS is a validated tool for quantifying AR symptoms and correlates well with the ARIA severity classification.^16^ The VAS consisted of a 100-mm horizontal line with verbal anchors at each end, where 0 indicated no symptoms and 100 indicated the worst possible symptoms. A VAS score of more than 50 mm indicated uncontrolled AR, 20 to 50 mm indicated partly controlled AR, and less than 20 mm indicated well-controlled AR. In the ARIA classification, mild AR was defined as answers of “no” to all 4 items, while a “yes” response to 1 or more items corresponded to moderate or severe AR.

The primary outcomes were between-group differences at day 180 in knowledge level, symptom control, medication adherence, and QOL. All outcomes were measured at baseline, 60 (±7) days (except for knowledge level), 120 (±7) days, and 180 (±7) days. Knowledge of INCS was assessed using a validated 4-item questionnaire with response options of “yes,” “no,” or “unsure.”^17^ Symptom control was evaluated using the Total Nasal Symptom Score (TNSS), assessing nasal congestion, sneezing, itching, and rhinorrhea on a scale of 0 to 3 (inverted score, 0 = severe; 1 = moderate; 2 = mild; 3 = none). The TNSS was measured across 2 time frames: (1) TNSS for the past 12 hours and (2) TNSS for the past 2 weeks. The TNSS for the past 12 hours captures volatile, day-to-day symptom fluctuations influenced by transient environmental exposures. The TNSS for the past 2 weeks reflects the mean symptom burden and is more sensitive to sustained behavioral changes from our educational intervention. This dual approach aligns with ARIA guidelines by capturing both immediate fluctuations and the effect of chronic disease.^18^

Medication adherence was measured using a diary booklet with a printed calendar, where participants marked the dates they administered INCS. Medication adherence outcomes were calculated as the number of days INCS were used during each follow-up period.

Participant’s QOL was captured by the survey using the European Quality of Life 5-Dimension 5-Level Instrument (EQ-5D-5L) and European Quality of Life Visual Analog Scale (EQ-VAS). The EQ-5D-5L consists of 5 dimensions: mobility, self-care, usual activities, pain or discomfort, and anxiety or depression. Each dimension offers 5 response levels: no difficulties, slight problems, moderate problems, severe problems, and extreme problems. The EQ-VAS captures the respondent’s overall health perception on a vertical VAS that takes values between 100 (indicating the best imaginable health) and 0 (indicating the worst imaginable health).^19,20^

### Statistical Analysis

Descriptive statistics were used to present the baseline characteristics of the 2 groups. Between-group comparisons were performed using the χ^2^ test (or the Fisher exact test) for categorical variables, the independent *t* test for normally distributed continuous variables, and the Mann-Whitney test for nonnormally distributed data. Normality was assessed using the Kolmogorov-Smirnov test. Statistical significance was defined as a 2-sided *P* < .05. All analyses were performed using the intention-to-treat (ITT) principle for all randomized patients. All primary outcomes were analyzed as coprimary end points with equal weighting, as no statistical hierarchy was predefined in the trial protocol.

Bayesian generalized linear mixed-effects models were applied using the brms package in R, version 4.2.2 (R Project for Statistical Computing) to compare continuous outcomes between the intervention and control arms with noninformative priors for all parameters due to the lack of preexisting data. Estimates derived on the noninformative prior were consistent with those obtained using the weakly informative prior (eTable 1 in Supplement 2). Different distributions were applied to address the specific characteristics of the outcome data. A hurdle gamma distribution was used for outcomes with zero inflation (eg, TNSS, EQ-VAS, and medication adherence), allowing for modeling both zero and positive values. A skew-normal distribution was applied to account for skewness, particularly in outcomes with zero and negative values (eg, EQ-5D-5L utilities). For nonnegative, left-skewed data (eg, knowledge levels), a gamma-like Gaussian distribution was used. Each model included fixed effects for treatment group and time points, as well as random intercepts to account for within-participant variability due to repeated measures. The posterior estimates for the regression coefficients and their 95% credible intervals (CrIs) were obtained using Markov chain Monte Carlo sampling with 4 chains and 2000 iterations, including a 1000-iteration burn-in period, for analyzing the primary end points. Meanwhile, knowledge level and medication adherence were analyzed using 4 chains and 8000 iterations, including a 2000-iteration burn-in period. Model convergence was accessed using trace plots and Rhat statistics. Good model convergence was deemed satisfactory when the Rhat value was less than 1.1 and the effective sample size relative to the total number of iterations exceeded 10%.^21^ Analyses were conducted using R, version 4.2.2 (R Project for Statistical Computing).

Missing data were addressed using last observation carried forward imputation. For confirmatory purposes, the primary outcome was analyzed using a per-protocol (PP) approach, which was restricted to participants with complete follow-up data.

Safety outcomes were analyzed through descriptive statistics, with between-group events comparisons performed using Poisson regression (SPSS, version 20.0; IBM Corp). Sample size calculations and power considerations are detailed in the trial protocol in Supplement 1.

## Results

**Figure 1.**
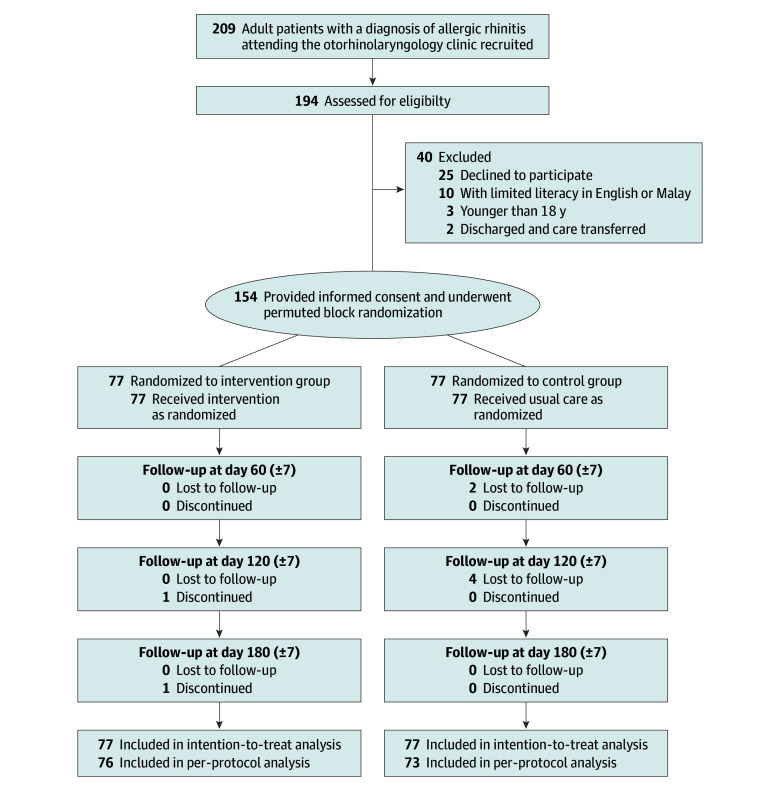
Recruitment and Flow of Participants

The study randomized 154 participants (intervention = 77; control = 77; mean [SD] age, 46.5 [17.0] years; 97 women [63.0%] and 57 men [37.0%]; 44 participants of Chinese ethnicity [28.6%], 27 participants of Indian ethnicity [17.5%], 81 participants of Malay ethnicity [52.6%], and 2 participants of other ethnicity [1.3%]), with 149 (96.8%) completing follow-up and 5 (3.2%) lost to follow-up (Table 1 and Figure 1). In the overall population, 75 participants (48.7%) had tertiary education; 72 participants (46.8%) were unemployed, retired, or students; and 60 of 82 participants (73.2%) worked fewer than 45 hours per week (Table 1). In addition, 80 participants (51.9%) reported food allergies, 112 (72.7%) reported allergies to other allergens, and 52 (33.8%) had pets at home. A total of 69 participants (44.8%) had a family history of allergic predisposition and 30 (19.5%) had comorbid asthma. Most participants used mometasone furoate (127 [82.5%]), followed by fluticasone furoate (24 [15.6%]) and budesonide (3 [1.9%]) for AR management.

**Table 1.** Participants’ Characteristics at Baseline

Variables	No. (%)		
	Overall (N = 154)	Intervention group (n = 77)	Control group (n = 77)
Age, mean (SD), y^a^	46.5 (17.0)	45.0 (17.0)	48.1 (16.9)
Sex			
Female	97 (63.0)	47 (61.0)	50 (64.9)
Male	57 (37.0)	30 (39.0)	27 (35.1)
Ethnicity			
Chinese	44 (28.6)	20 (26.0)	24 (31.2)
Indian	27 (17.5)	11 (14.3)	16 (20.8)
Malay	81 (52.6)	44 (57.1)	37 (48.1)
Other (eg, Punjabi)	2 (1.3)	2 (2.6)	0
Marital status			
Single	41 (26.6)	25 (32.5)	16 (20.8)
Married	111 (72.1)	52 (67.5)	59 (76.6)
Divorced	2 (1.3)	0	2 (2.6)
Highest educational level			
Primary	12 (7.8)	6 (7.8)	6 (7.8)
Secondary	67 (43.5)	36 (46.8)	31 (40.3)
Tertiary	75 (48.7)	35 (45.5)	40 (51.9)
Occupation			
Managerial	42 (27.3)	23 (29.9)	19 (24.7)
Intermediate and small employer	18 (11.7)	10 (13.0)	8 (10.4)
Lower supervisory, technical, semiroutine, or routine	22 (14.3)	13 (16.9)	9 (11.7)
Unemployed, retired, or student	72 (46.8)	31 (40.3)	41 (53.2)
Work h/wk^b^			
<45	60 (73.2)	36 (78.3)	24 (66.7)
≥45	22 (26.8)	10 (21.7)	12 (33.3)
Smoking status			
Never smoked	140 (90.9)	69 (89.6)	71 (92.2)
Current smoker	7 (4.5)	4 (5.2)	3 (3.9)
Former smoker	7 (4.5)	4 (5.2)	3 (3.9)
Pets at home	52 (33.8)	25 (32.5)	27 (35.1)
Food allergy	80 (51.9)	35 (45.5)	45 (58.4)
Allergy to other allergens	112 (72.7)	55 (74.0)	57 (74.0)
History of nasal surgery	12 (7.8)	6 (7.8)	6 (7.8)
Family history of allergy	69 (44.8)	37 (48.1)	32 (41.6)
Comorbidity of asthma	30 (19.5)	17 (22.1)	13 (16.9)
INCS prescribed at baseline			
Mometasone furoate 50 µg	127 (82.5)	64 (83.1)	63 (81.8)
Fluticasone furoate 27.5 µg	24 (15.6)	11 (14.3)	13 (16.9)
Budesonide 64 µg	3 (1.9)	2 (2.6)	1 (1.3)
Oral antihistamine			
Loratadine 10 mg	123 (79.9)	63 (81.8)	60 (77.9)
Others (eg, levocetrizine)	31 (20.1)	14 (18.2)	17 (22.1)

Abbreviation: INCS, intranasal corticosteroid.

^a^ Normality assumptions were met. The Kolmogorov-Smirnov test indicated no significant deviation from normality for the intervention group (*P* = .07) and the control group (*P* = .18).

^b^ There were 82 participants overall, 46 in the intervention group and 36 in the control group.

The consistency between the ITT and PP analyses suggests that the missing data had a negligible effect on the outcomes, except for TNSS for the past 2 weeks (eTables 2 and 3 in Supplement 2). The percentage of missing data was 1.3% (2 of 154) at 60 (±7) days, 3.2% (5 of 154) at 120 (±7) days, and 0.6% (1 of 154) at 180 (±7) days. Participants with and participants without missing data were comparable (eTable 4 in Supplement 2). The Little MCAR (missing completely at random) test indicated that data were missing completely at random.

Table 2 presents the longitudinal clinical characteristics of AR in the 2 study arms. A significant finding was observed in the VAS category, indicating that with each increase in the number of participants in the intervention group, there was a decrease in the log odds of being in the poor VAS category (estimate [SE], −0.77 [0.25]; 95% CI, −1.26 to −0.28). Similarly, in determining the severity of AR, with each participant who received the intervention, the log odds of being classified in the mild AR category compared with the moderate-to-severe AR category significantly increased (estimate [SE], 0.62 [0.24]; 95% CI, 0.15-1.08).

**Table 2.** Clinical Characteristics of AR

Variables	No. (%)								Between-group differences, estimate (SE) [95% CI]
	Baseline		Day 60 (±7)		Day 120 (±7)		Day 180 (±7)		
	Intervention (n = 77)	Control (n = 77)	Intervention (n = 77)	Control (n = 77)	Intervention (n = 77)	Control (n = 77)	Intervention (n = 77)	Control (n = 77)	
VAS, median (IQR), mm	30.0 (7.5-54.0)	50.0 (10.5-64.0)	NA	NA	NA	NA	0.0 (0.0-19.5)	17.0 (0.0-50.0)	NA
Good	27 (35.1)	21 (27.3)	NA	NA	NA	NA	58 (75.3)	39 (50.6)	−0.77 (0.25) [−1.26 to −0.28]^a^^,^^b^
Moderate	20 (26.0)	12 (15.6)	NA	NA	NA	NA	11 (14.3)	24 (31.2)	
Poor	30 (39.0)	44 (57.1)	NA	NA	NA	NA	8 (10.4)	14 (18.2)	
ARIA classification									
Severity of AR									
Mild	44 (57.1)	37 (48.1)	55 (71.4)	54 (70.1)	60 (77.9)	45 (58.4)	61 (79.2)	44 (57.1)	0.62 (0.24) [0.15 to 1.08]^b^^,^^c^
Moderate to severe	33 (42.9)	40 (51.9)	22 (28.6)	23 (29.9)	17 (22.1)	32 (41.6)	16 (20.8)	33 (42.9)	
Frequency of AR									
Intermittent	51 (66.2)	45 (58.4)	59 (76.6)	58 (75.3)	58 (75.3)	54 (70.1)	63 (81.8)	53 (68.8)	0.36 (0.25) [−0.14 to 0.86]^c^
Persistent	26 (33.8)	32 (41.6)	18 (23.4)	19 (24.7)	19 (24.7)	23 (29.9)	14 (18.2)	24 (31.2)	

Abbreviations: AR, allergic rhinitis; ARIA, Allergic Rhinitis and its Impact on Asthma; NA, not applicable; VAS, visual analog scale of AR severity.

^a^ Analyzed with generalized linear mixed-effects models using a cumulative link mixed model fitted with the Laplace approximation. The control group served as the reference for the independent variable, while the poor VAS category served as the reference for the dependent variable.

^b^ Indicates a statistically significant difference between the intervention and control groups.

^c^ Analyzed with generalized linear mixed-effects models. Between-group differences in the intervention vs control group were analyzed using binary logistic regression, with the control group set as the reference. “Moderate-to-severe” served as reference for the dependent variable for severity of AR. “Persistent” served as reference for the dependent variable for frequency of AR.

### Primary Outcomes

#### Knowledge Level

**Figure 2.**
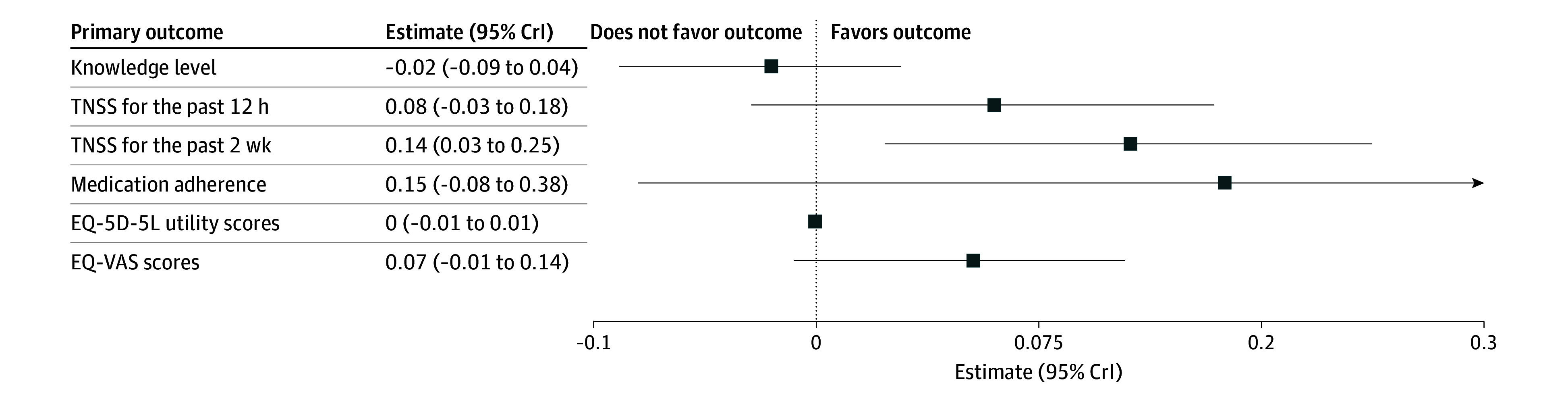
Forest Plot Displaying the Regression Coefficient Estimates and 95% Credible Intervals (CrIs) for the Primary Outcomes EQ-5D-5L indicates European Quality of Life 5-Dimension 5-Level Instrument; EQ-VAS, European Quality of Life Visual Analog Scale; and TNSS, Total Nasal Symptom Score.

**Figure 3.**
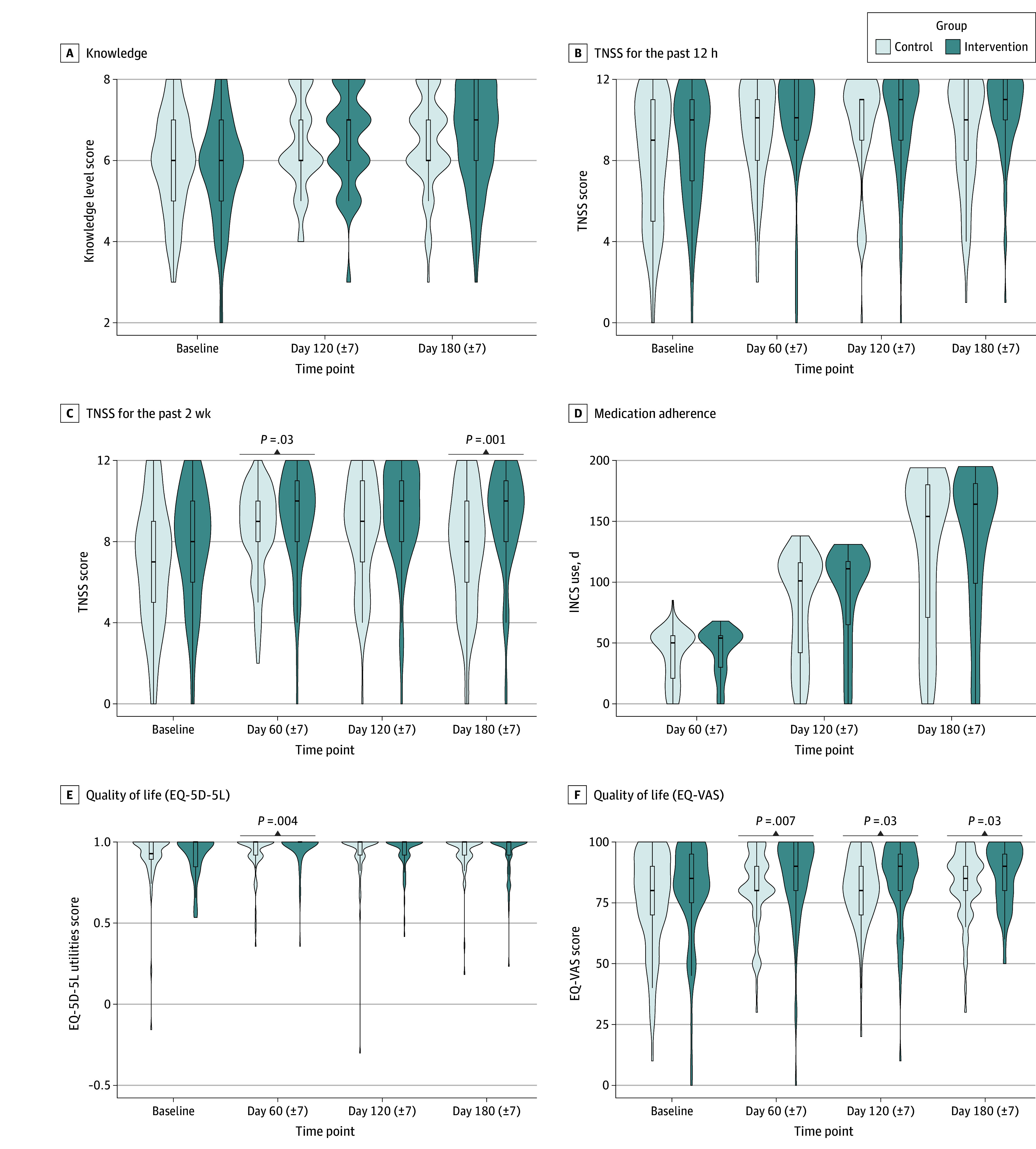
Violin Plots of Primary Outcomes Violin plots show the distribution density by group across time points. Box plots display median (center line) and IQR (box); whiskers indicate 1.5 times the IQR. *P* values were analyzed using the Mann-Whitney test. EQ-5D-5L indicates European Quality of Life 5-Dimension 5-Level Instrument; EQ-VAS, European Quality of Life Visual Analog Scale; INCS, intranasal corticosteroid; and TNSS, Total Nasal Symptom Score.

No significant between-group differences in knowledge levels were observed (Figure 2). However, the intervention group demonstrated narrower distributions and higher median scores (Figure 3A).

#### Symptom Control

Symptom control was evaluated and compared between the intervention and control groups using the TNSS for the past 12 hours and the TNSS for the past 2 weeks. For the TNSS for the past 12 hours, the intervention group exhibited higher median TNSSs at all time points, with narrower distributions, suggesting reduced variability in symptom severity (Figure 3B). This median TNSS may indicate a clinical benefit, although it did not reach statistical significance compared with the control group (estimate [SE], 0.08 [0.05]; 95% CrI, −0.03 to 0.18) (Figure 2).

For the TNSS for the past 2 weeks, the AR-PRISE intervention demonstrated a statistically significant effect compared with the control group (estimate [SE], 0.14 [0.06]; 95% CrI, 0.03-0.25) (Figure 2). Significant reductions in the TNSS for the past 2 weeks were observed at all time points, including day 60 (estimate [SE], 0.18 [0.05]; 95% CrI, 0.08-0.28), day 120 (estimate [SE], 0.21 [0.05]; 95% CrI, 0.11-0.31), and day 180 (estimate [SE], 0.11 [0.05]; 95% CrI, 0.01-0.21) (eTable 2 in Supplement 2). Violin plots (Figure 3C) visually supported these findings, illustrating consistently higher median TNSSs and narrower distributions in the intervention group.

#### Adherence

Medication adherence demonstrated progressive improvement throughout the study period, with the intervention group exhibiting a higher number of median adherence days (Figure 3D). However, these between-group differences were not statistically significant (Figure 2).

#### Quality of Life

No statistically significant differences were found between the groups in EQ-5D-5L utility scores or the EQ-VAS (Figure 2). However, the intervention group showed significantly improved EQ-5D-5L utility scores at day 60 (±7) (Figure 3E) and demonstrated consistent EQ-VAS improved performance across all follow-ups, with higher median scores and better density distributions compared with controls (Figure 3F). A significant positive association between time and EQ-VAS was observed, indicating progressive improvement during the intervention period (eTable 2 in Supplement 2).

#### Safety

The control group had 1.82 (95% CI, 1.14-2.90) times higher odds of experiencing INCS-related adverse effects compared with the intervention group (eTable 5 in Supplement 2). No significant differences between the groups were found for unscheduled health facility visits, number of other medications taken, use of traditional and complementary products or procedures, or oral antihistamine adverse effects.

## Discussion

Our study demonstrated that the AR-PRISE intervention significantly improved AR symptom control (TNSS for the past 2 weeks) over 6 months. The intervention group had higher log odds of having mild AR than moderate-to-severe AR compared with the control group. These findings align with prior evidence emphasizing the importance of structured patient education and self-management in chronic disease management.^22,23^ By adapting key elements of the Chronic Care Model,^24^ AR-PRISE positions pharmacists as integral players in AR care, promoting both improved clinical outcomes and patient empowerment.

The AR-PRISE intervention showed a stronger treatment effect observed in the TNSS for the past 2 weeks (sustained control) vs the TNSS for the past 12 hours (immediate symptoms), reflecting its focus on long-term disease management. Nevertheless, because potential waning effects or behavioral drift cannot be ruled out,^25^ its long-term sustainability remains uncertain. Future studies should examine whether incorporating booster education sessions could sustain the intervention’s benefits beyond 180 days.

In addition to symptom control, the intervention group demonstrated a significantly lower likelihood of experiencing poor VAS scores and moderate-to-severe AR compared with the control group. This finding is particularly impactful as it highlights the intervention’s role in preventing escalation to more severe disease states. This improvement was likely driven by the intervention’s emphasis on correct INCS use and patient education, as INCS efficacy depends heavily on proper administration techniques.^26^ Not only does the correct use of INCS enhance its efficacy, but it also appears to reduce the likelihood of adverse effects, as supported by our findings. In addition, the model’s patient-centered approach may have improved early symptom recognition and timely action, contributing to fewer episodes of severe AR.

However, the lack of statistically significant differences in knowledge, despite symptom reduction, suggests additional strategies are needed for behavioral change. One possible explanation is the narrow focus of the knowledge questionnaire, which assessed only INCS-related knowledge. Future studies could adopt broader measures to capture the full spectrum of knowledge gained from educational interventions. In addition, the intervention was delivered through a combination of 1-time face-to-face counseling and video education, which was accessible throughout the follow-up period. However, there was no reinforcement or monitoring to ensure patients revisited the video content, which may have contributed to the lack of significant changes in knowledge. Incorporating periodic follow-up sessions or actively encouraging patients to revisit the educational video could improve knowledge retention and engagement.^27^

Medication adherence showed no significant improvement in the intervention group despite targeted education on proper medication use. This finding aligned with findings of the meta-analysis by Wiecek et al^28^ that education alone is insufficient for sustaining adherence, whereas multicomponent interventions addressing cognitive, behavioral, and affective domains yield superior outcomes. Future AR-PRISE iterations should adopt the World Health Organization Multidimensional Adherence Model, targeting socioeconomic, patient, condition, therapy, and system factors simultaneously.^29^

### Limitations

This study has some limitations. The single-center design and exclusion of non-English or Malay speakers may limit generalizability, although our cohort’s racial and ethnic distribution aligned with national demographics.^30^ Future multicenter trials are needed to confirm broader applicability. The discrepancy between the PP and ITT results may reflect reduced sample size and lower statistical power in the PP analysis. However, we prioritized the ITT findings to preserve randomization integrity.

Disease-specific tools, such as the Rhinoconjunctivitis Quality of Life Questionnaire, may better capture AR-specific QOL changes (eg, sleep disturbances and nasal symptoms) than generic measures.^31^ However, to our knowledge, no validated Malay translation of the Rhinoconjunctivitis Quality of Life Questionnaire currently exists, unlike the EQ-5D-5L, which has been formally adapted and validated for Malaysian populations.

## Conclusions

In this randomized clinical trial of adults with AR, the AR-PRISE intervention showed statistically significant improvement in sustained symptom control (TNSS for the past 2 weeks) compared with standard care over 180 days, although its effects on immediate symptom assessment (TNSS for the past 12 hours) were not statistically significant. However, no significant differences were observed for coprimary outcomes, including knowledge levels, medication adherence, or QOL. By leveraging pharmacists’ accessibility and expertise, this model promotes a team-based approach to AR management. Future studies should focus on optimizing the AR-PRISE model to enhance knowledge retention, adherence, and sustainability while exploring its applicability in other chronic conditions.

## Notes

Supplement 1. Trial Protocol and Statistical Analysis Plan

Supplement 2. **eTable 1.** Estimates of Prior Distributions for Each Primary End Point **eTable 2.** Primary End Points for Efficacy Evaluation **eTable 3.** Participants’ Primary Outcomes (PP vs ITT) **eTable 4.** Demographic Characteristics of Study Dropouts Versus Completers **eTable 5.** Safety

Supplement 3.
Data Sharing Statement
