# Supplementary material for: Pharmacist-Led Education Intervention for Adults With Allergic Rhinitis: A Randomized Clinical Trial
Source: JAMA Netw Open. 2025 Jul 16;8(7):e2517160. doi: 10.1001/jamanetworkopen.2025.17160 (PMC12268493; doi:10.1001/jamanetworkopen.2025.17160)
Supplement: Supplement 1. — Trial Protocol and Statistical Analysis Plan [file jamanetwopen-e2517160-s001.pdf]

1 GENERAL INFORMATION

Study Title:

A prospective, randomized, controlled, parallel group, single-centre trial to assess the effectiveness of Pharmacist-led educational protocol in patients with Allergic Rhinitis in a Tertiary Hospital

Protocol number, version number and date:

Protocol No.: STAR, Version 3.0, dated 30 Jun 2023

1. Chew Chii Chii
   Clinical Research Centre, Hospital Raja Permaisuri Bainun, Ipoh, Ministry of Health, Malaysia

2. Dr. Lim Xin Jie
   Clinical Research Centre, Hospital Raja Permaisuri Bainun, Ipoh, Ministry of Health, Malaysia

3. Chan Huan Keat
   Clinical Research Centre, Hospital Sultanah Bahiyah, Alor Setar, Ministry of Health, Malaysia

4. Dr. Doris George
   Pharmacy Department, Hospital Raja Permaisuri Bainun, Ipoh, Ministry of Health, Malaysia

5. Dr. Pathma Letchumanan
   Department of Otorhinolaryngology
   Hospital Raja Permaisuri Bainun, Ipoh, Ministry of Health, Malaysia

6. Dr. Philip Rajan
   Department of Otorhinolaryngology & Clinical Research Centre, Hospital Raja Permaisuri Bainun, Ipoh, Ministry of Health, Malaysia

7. Dr. Kelvinder Singh A/L Awtar Singh
   Department of Otorhinolaryngology & Clinical Research Centre, Hospital Raja Permaisuri Bainun, Ipoh, Ministry of Health, Malaysia

8. Dr. Loong Siow Ping
   Department of Otorhinolaryngology & Clinical Research Centre, Hospital Raja Permaisuri Bainun, Ipoh, Ministry of Health, Malaysia

Name of Sponsor: No external funding

Source of Funding: None declared

Study site/s: Hospital Raja Permaisuri Bainun, Ipoh

Declaration of conflict of interest: The members of the study team declare no competing interests.

SYNOPSIS

**Title of study**	A prospective, randomized, controlled, parallel group, single-centre trial to assess the effectiveness of Pharmacist-led educational protocol in patients with Allergic Rhinitis in a Tertiary Hospital
**Sponsor**	No external funding
**Investigators**	Pharmacists, medical officer, consultant rhinologists and consultant otolaryngologists
**Study period**	12 months
	Planned date of first subject enrolment: 1st Jan 2023<br>Planned date of last subject completed: 30th June 2024
**Objectives**	To determine the effectiveness of a pharmacist-led educational protocol compared to standard pharmaceutical care with respect to patients diagnosed with allergic rhinitis in a tertiary hospital.
**Methods**	This is a randomised, controlled, parallel group design study. This study will be conducted at Ear, Nose and Throat (ENT) clinic at Hospital Raja Permaisuri Bainun, Perak State of Malaysia.
**Number of centres**	One
**Inclusion criteria**	1. Patient who is Malaysian, aged 18 to 80, with medical diagnoses of moderate or severe allergic rhinitis according to ARIA guidelines, who attended an ENT outpatient clinic in the period of study, and<br>2. capable of reading and writing in English or Malay
**Exclusion criteria**	1. Pregnant or lactating mothers.<br>2. Patients who had comorbid diagnosis of chronic rhinosinusitis.<br>3. Patients who had psychiatric problems or dementia, at the discretion of the physician, were deemed unfit to participate in this study.<br>4. Patients with terminal illnesses, at the discretion of the physician, are unfit to participate in this study.<br>5. Patients with morbidities or diseases, at the discretion of the physician, are unfit to participate in this study.<br>6. Patients with post-covid conditions with symptoms that continue beyond 3 months after being infected, at the discretion of the physician, are unfit to participate in this study
**Intervention group**	Patients receive pharmacist-led educational protocol and standard pharmaceutical care (receive medications from the pharmacy after being seen by a medical doctor in the ENT clinic)
**Control group**	Patients receive standard pharmaceutical care (usual health services of that patients will be receiving their medication at the counter of outpatient pharmacy in the hospital)
**Criteria for evaluation**	Primary criteria. The differences between the intervention and control groups in the aspect of<br>1. knowledge of intranasal corticosteroid<br>2. Symptom control<br>3. adherence to intranasal corticosteroid administration<br>4. quality of life
**Safety parameters**	• Patients with allergic rhinitis symptoms not adequately control are advised to walk-in to the ENT clinic without waiting for the appointment date.

	• Patients with allergic rhinitis co-exist asthma are advise to go emergency department if the asthma condition is uncontrolled.
**Statistical methods**	Sample size and power considerations<br>Control group: 70 participants<br>Intervention group: 70 participants<br>Consider 10% dropout rate, 77 participants are required from each group. 154 participants will be recruited.

LIST OF ABBREVIATIONS

Abbreviations	Full term
AR	Allergic rhinitis
CRF	Clinical Report Form
ENT	Ear, nose and throat
°C	Degree Centigrade
QoL	Quality of life
TNSS	Total nasal symptom score

2 BACKGROUND & LITERATURE REVIEW

Sneezing, nasal congestion, nasal itching, and rhinorrhoea are signs of allergic rhinitis (AR), which is caused by inflammation of the nasal membranes (Bousquet et al., 2020). The prevalence of AR occurs at a rate of 10 to 30% of adults (World Allergy Organization (WAO), 2013). In the USA and Europe, the burden of AR has increased in the last two decades, with a prevalence of 10 to 20% (Ozdoganoglu and Songu, 2012). The prevalence of AR in Asia varies considerably depending on the geographical location (Chong and Chew, 2018). In Malaysia, the prevalence of AR was reported as ranged from 21 to 24% in adult patients (Lim et al., 2015).

The risk factors triggering allergic rhinitis have been changing over time, and these factors are different between Western and Asian countries. The common risk factors in both regions are pet adoption and family history. In Asian countries, demographic factors like age, gender, race, and nationality are linked to a higher risk of AR.(Chong and Chew, 2018).

Because of the significant prevalence of allergic rhinitis and its impact on quality of life, it has been designated as a major chronic respiratory disease (Bousquet et al., 2001). Allergic rhinitis can have a major impact on quality of life (Tripathi and Patterson, 2001), decreasing sleep and negatively impacting leisure, social life, school performance (Simons, 1996), and work productivity (Blanc et al., 2001). Allergic rhinitis has significant direct and indirect financial implications. Indirect costs include sick leave, school and job absences, and productivity loss (Sullivan and Weiss, 2001).

In the United States, the total direct medical cost of AR was 3.4 billion USD. In Europe, direct healthcare costs are comprised of €159 to €554 per patient per year in treating AR. In the Asian countries, including Turkey and India, direct healthcare costs were ranged from 79 to 215 USD per individual per year. In Korea, a total direct cost of 224 USD million was calculated (Dierick et al., 2020). Given its high prevalence, the management of AR is a costly disease to treat on a population basis.

A recent study shows that there are 3 significant factors influencing patients in managing their own AR: (i) general practitioners, (ii) pharmacists, and (iii) patients' own experience (Cvetkovski et al., 2018). An integrated care pathway (ICP) involving a multidisciplinary team including both medical doctors and pharmacists is recommended by the Allergic Rhinitis and its Impact on Asthma (ARIA) guidelines (Bousquet et al., 2019). It has been shown that when pharmacists work with patients to assist them in setting their goals for AR management, the long-term outcomes are better (Cvetkovski et al., 2020).

Role of pharmacist in managing allergic rhinitis

The role of pharmacists in AR management has been reiterated by the international clinical practise guidelines and literature (Arsoy et al., 2018; Bousquet et al., 2019). Pharmacists resolve medication-related problems, optimise regimens, and provide medication information (Arsoy et al., 2018; Lourenço et al., 2020).

In Malaysia, pharmacists play important roles in government-funded health institutions such as hospitals and primary care clinics (Jaafa et al., 2013). Typically, pharmacists in the local government-funded health institutions may have been limited to intranasal corticosteroid spray demonstrations and counselling. The roles of pharmacists could be expanded to include non-pharmacological management, such as educating patients on the aspects of allergen identification and avoidance of exposure (Arsoy et al., 2018; Bridgeman, 2017; Lourenço et al., 2020).

The impact of pharmacist-led interventions

Pharmacists who have been trained for AR management reported that they have taken initiatives to communicate with patients in AR medicine selection and intranasal inhaler administration while patients reported having good control of symptoms in AR. (Arsoy et al., 2018; Cvetkovski et al., 2020).

Studies with regard to pharmacist-led intervention have been limited. There were only four similar studies identified. Of these four, three studies were identified via a systematic review in which these studies were conducted between 2000 and 2019, with common limitations of small sample size (n = 47 to 63). The interventions introduced include educational interventions conducted using a randomised controlled trial, pharmacist-goal setting vs patient-goal setting using an approach of mixed-method, and the last study was a case study that observed the impact of pharmaceutical care. All three studies show significant improvement in symptom control and quality of life following pharmacist-led interventions. The author summarised that there is a need for more research on pharmacy-developed practises and interventions to determine AR measurable outcomes in patients (José et al., 2020). The forth study was conducted recently 2020 shows that improvement in quality of life among patients who received pharmacist-led intervention was higher than those who received standard care, as seen in two recent studies (Smith et al., 2020).

A pharmacist-led educational protocol

The management of AR could be strengthened by the development of strategies, tools, and policies (Cvetkovski et al., 2019). The development of a local standard of care for patients that considers pharmacy practice, infrastructure, workforce, and regulation is highly recommended and advocated by the international guidelines (Bosnic-Anticevich et al., 2019; Bousquet et al., 2004, 2004). Currently, there is a lack of local pharmacist-led AR management protocols in place and the existing international guidelines are mainly focused on the practise of community pharmacists (Arsoy et al., 2018; Bosnic-Anticevich et al., 2019; Bousquet et al., 2019).

Moreover, patients complain about conflicting and fragmented information delivered by different healthcare providers (Cvetkovski et al., 2020). Development of an education protocol would standardise communication among healthcare providers when delivering messages to patients. Therefore, a pharmacist-led educational protocol should include standard educational or counselling points with regards to the disease nature, choice of pharmacotherapy, expectations of treatments, allergen identification and avoidance, and intranasal corticosteroid administration technique. The educational points should be structured such that they allow other professions, such as physicians and nurses, to use this protocol when providing information in simple language to the patients.

A pharmacist-led educational protocol not only helps patients understand AR management but may also serve as a general guide for healthcare providers to learn about this disease management. This would enable healthcare providers to deliver concordant care in a standardised manner in an effort to achieve medication adherence and attain improved clinical outcomes (Meltzer et al., 2017).

Problem statement

With approximately one-in-five of the population having AR, the burden of allergic rhinitis on the health system and the workload of healthcare providers is substantial. Within the context of physicians' busy schedules, detailed counselling by a physician for each patient is unlikely. Pharmaceutical care management by a pharmacist has been advocated by the Pharmacy ARIA guideline and literatures. A study shows that pharmacists can help patients with AR control their symptoms and improve their quality of life through educational interventions (Arsoy et al., 2018). The roles of pharmacists should be expanded to include non-pharmacological counselling such as allergen avoidance and identification (Arsoy et al., 2018).. However, locally, the role of pharmacists in the public service has been limited to intranasal corticosteroid spray counselling.

The reported adherence rate to intranasal corticosteroids ranged from 32.7 to 53.2% in allergic rhinitis treatments. The majority of local healthcare providers stated that treatment efficacy (81.8%) and adverse effects (83.8%) affect medication compliance and these problems remain unaddressed (Abdullah et al., 2020). Health literacy is an important component of adherence to medications (Bousquet et al., 2020). Often, medication is not supplemented by the appropriate education of allergy, trigger, and symptoms identification & management of the disease (Yawn, 2008). Studies show that knowledge about AR and its management remains low, which has widely ranged from 22 to 70.1% to intranasal corticosteroid (Hellings et al., 2012; Retinasekharan et al., 2021). Nonadherence to intranasal corticosteroid could result in increased healthcare burden, including poor clinical outcomes, a rise in comorbid diseases, higher costs, and death (Chisholm-Burns and Spivey, 2012).

The management of AR could be strengthened by the development of strategies, tools, and policies (Cvetkovski et al., 2019). The development of a local standard of care for patients that considers pharmacy practice, infrastructure, workforce, and regulation is highly recommended and advocated by the international guidelines (Bosnic-Anticevich et al., 2019; Bousquet et al., 2004, 2004). Currently, there is a lack of local pharmacist-led AR management protocols in place and the existing international guidelines are mainly focused on the practise of community pharmacists (Arsoy et al., 2018; Bosnic-Anticevich et al., 2019; Bousquet et al., 2019).

Moreover, patients complain about conflicting and fragmented information delivered by different healthcare providers (Cvetkovski et al., 2020). Development of an education protocol would standardise communication among healthcare providers when delivering messages to patients. Therefore, a pharmacist-led educational protocol should include standard educational or counselling points with regards to the disease nature, choice of pharmacotherapy, expectations of treatments, allergen identification and avoidance, and intranasal corticosteroid administration technique. The educational points should be structured such that they allow other professions, such as physicians and nurses, to use this protocol when providing information in simple language to the patients.

Inadequate awareness about ARIA guidelines among healthcare providers has been reported (Canonica et al., 2015; Irwani Ibrahim, Bao Li Chew, Wai Wai Zaw, 2014). Notably, in view of different healthcare systems across different countries, the ARIA pharmacy guideline encourages the creation of local standards of care for managing AR patients

(Lourenço et al., 2020). A lack of local standardised protocol for healthcare providers to convey critical information to patients may result in patients receiving fragmented information.

Potential Risks

A pharmacist-led educational protocol not only helps patients understand AR management but may also serve as a general guide for healthcare providers to learn about this disease management. This would enable healthcare providers to deliver concordant care in a standardised manner in an effort to achieve medication adherence and attain improved clinical outcomes (Meltzer et al., 2017).

The foreseeable risk to the participants is minimal. The participant who will be assigned at random to either the control or intervention group will be receiving usual care while the intervention group will be receiving a pharmacist-led educational protocol. Physically, there is no investigational product in this study, the participants will not be exposed to any new substances.

The participants will be asked for their demographic details, and they will be asked to self-administer a validated questionnaire assessing their knowledge with regards to intranasal corticosteroid, symptom control, and quality of life. Additionally, they will be given a diary card to record their medication usage. None of the tools used involved any sensitive questions or invasive procedures. The questionnaire administration process and daily recording of medication intake will not affect the participants from psychological, social, legal, or economic perspectives.

The participants will be followed up on day 60±7, 120±7 and 180±7. They will need to allocate some time to answer the questionnaire. The follow-up could be conducted in the form of a physical visit to the clinic should they be scheduled for a doctor's appointment, or they will be followed up virtually using Zoom or Google Meet. No additional expenses will be incurred for the participants. The need for participant follow-up is important to determining the long-term sustainability of knowledge and behaviour change as reflected in the outcomes (symptom control and quality of life) after receiving a pharmacist-led education protocol versus those receiving usual care.

Shortening the duration of follow-up for the participants has been considered. However, in the consideration of the robustness of the pharmacist-led educational protocol, up to 180 days of follow-up would increase the overall effectiveness of the research effort to better convince health policymakers to adapt this protocol for clinical practice (Salkind, 2022).

Potential Benefits

The conduct of this randomised controlled trial will be important to prove the effectiveness of a pharmacist-led educational protocol. If this protocol is shown to be effective in improving clinical outcomes and participant quality of life, the organisation will have clearer guidance for pharmacists in providing counselling points to patients with allergic rhinitis. Should this protocol be implemented in clinical practice, the "education" section of this protocol can be used as a standard communication tool such that all of the healthcare providers, including the doctor, give the same advice to the patients. This would address the common problems faced by patients who complain of receiving fragmented and conflicting information from different healthcare providers (Cvetkovski et al., 2020).

Patients who receive adequate information about the nature of the disease, pharmaceutical treatment, and treatment expectations change their behaviour in disease management and medication adherence (Lourenço et al., 2020). This would potentially improve their symptom control and quality of life. This in turn will reduce the healthcare burden in terms of clinic visits, cost, and comorbidities.

3 OBJECTIVES

Primary Objective

To determine the effectiveness of the pharmacist-led educational protocol in managing allergic rhinitis between a control group of patients who are receiving standard pharmaceutical care and an interventional group of patients receiving an educational interventional protocol plus standard pharmaceutical care, based on the following specific parameters: -

1. Knowledge of intranasal corticosteroids
2. Symptom control
3. Medication adherence
4. Quality of life

Secondary Objective

To determine within group difference for the 4 primary parameters (knowledge, symptom control, medication adherence, quality of life)

Societal value or beneficial outcome of study

Should the pharmacist-led protocol proven to be effective in improving knowledge, symptom control, medication adherence and quality of life in allergic rhinitis patients, this protocol is potentially implemented in clinical practice, which in turn would reduce the burden of the ENT outpatient clinic, as well as reduce the healthcare setting burden.

4 ETHICS & REGULATORY CONSIDERATIONS

Ethical conduct of the study

Ethical approval will be obtained from the Medical Research and Ethics Committee (MREC) through NMRR before the study is conducted.

The study will be conducted in compliance with the protocol and standard operating procedures. These are designed to ensure adherence to the ethical principles that have their origin in the "World Medical Association Declaration of Helsinki", "Malaysian Guidelines for Good Clinical Practice" and applicable regulatory Requirements.

All study data will be stored for 3 years after the study findings are published and then destroyed. All data will be kept under lock and key in the office cabinet and only accessible by the researchers. Participants in this study will not be given accessed nor informed of the findings' outcome. The participants will be informed with the new information if it becomes available during the study. The patient educational material will be shared with all the participants, including the control group, at the end of the study.

Participants can choose to withdraw at any time. The physician or investigator may withdraw participants if there is an associated risk with the participants’ continuing participation in this study. All withdrawn participants will be reminded to continue receiving their standard medical care at the ENT clinic at Hospital Raja Permaisuri Bainun. Withdrawn participants will not be replaced. The data collected from the withdrawn participants will be included in the analysis.

The participants will not be informed individually about the study findings; however, the patient education material will be shared with all the participant (including the control group)

at the end of this study. The control group participants will be subjected to structured pharmacist counselling at the end of study, right after their data are collected. All the information obtained from this study will be kept confidential and only summarized data, without unique identifier, will be presented in reports or publications upon getting permission from the Director General of Health, Ministry of Health Malaysia.

Informed consent and subject information

Written informed consent will be obtained from every participant prior to participation in this study. The investigator will inform every participant in detail about the nature of the study, its purpose, and the probability of random assignment to intervention groups, those aspects of the study that are experimental, the procedures involved, the reasonably expected benefits, the expected duration of participation will be 6 months and the approximate number of 154 participants will be involved.

A copy of the Patient Information Sheet and signed Consent Form will be given to the participants (Appendix A). The original copy will be filed by the principal investigator in the Investigator's Study File.

The participants will be informed that

- Their participation in this study is voluntary and that they may withdraw from this study at any time for any reason and that withdrawal of consent will not affect their subsequent medical treatment or relationship with the treating physician.
- They will be notified in a timely manner if information becomes available that may be relevant to their willingness to continue participation in the study.
- The risk of participating in this study is minimal, should any injuries occurred; they will be advised to come to the ENT clinic or emergency department immediately. Tentatively, no additional cost is anticipated to be incurred to the patients.
- This study does not receive any external funding; the participants will not be paid.
- Any foreseeable circumstances and/or reasons by the discretion of the physician under which the participants' participation in the study may be terminated.
- The principle investigator to contact for further information regarding the study and whom to contact in the event of study related injury.

The place to obtain written informed consent will be conducted at a designated meeting room that is empty during clinic day and it is considered sufficiently private for the participants and investigator, which is located in the ENT outpatient clinic. The timing to take written informed consent is set right after patients have attended their routine visit to seek medical consultation with the medical doctor.

Patient protection procedures

Procedures in the event of Emergency

In the event of emergency, the participants are advice to go the emergency department, Hospital Raja Permaisuri Bainun or to their nearest emergency department of any government-funded hospitals.

If participants' allergic rhinitis symptoms are not adequately controlled, they are advised to walk-in to the ENT clinic without having to wait for the appointment date.

Procedures in the event of Pregnancy

The patients will be instructed to inform the investigators and physician if she becomes pregnant during the study. Should the participants become pregnant, she will be discontinued from the study. Since there is no investigational product is involved, the participants will not be follow up thereafter.

Patient data protection

The investigator will be responsible to assure that the participants' anonymity to be maintained and that the confidentiality of records and documents that could identify participants will be protected, respecting the privacy and confidentiality rules in accordance with applicable regulatory requirements. Individuals involved in this study for medical care, qualified monitors and auditors, and governmental or regulatory authorities may inspect and copy the medical records, where appropriate and necessary. All participants will not be given access to their personal information and study data.

- The participants will be identified only by their assigned participants' identification number in all clinical report forms (CRF), study-related records and documents. There will be a Subject Identification List (Appendix B) with information on name,

contact number, address and hospital registration number with the corresponding study identification number. This Subject Identification List will be kept by the principle investigator on site in a locked-cabinet.

- All patients who are screened will be recorded in the Screening and Enrolment Log, which includes screen failures (Appendix C). This list will be kept separately in the study investigation file and separate from data collection sheet.
- Signed informed consent will be kept in a locked-cabinet, which can only be accessed by investigators on site.
- All data will be entered into and SPSS version 20.0 and the file will be password-protected. The electronic database only allows assigned investigators to enter and view data.

5 STUDY DESIGN

Study endpoints

The primary and secondary endpoints of this study are shown in table 1

Table 1: study endpoints

**Primary endpoint**	Descriptions
1. Knowledge of intranasal corticosteroid	The mean scores of participants who correctly answer each question in the control and intervention groups with regard to the intranasal corticosteroid.
2. Symptom control	The mean difference in symptom control between the control and interventional groups.
3. Medication adherence	The mean adherence rate difference for the number of doses of intranasal corticosteroid spray taken by the participant between the control and interventional groups.
4. Quality of Life	The mean difference in quality of life between the control and interventional groups.
**Secondary endpoint**	Within group changes from Day 0, to Day 60±7, Day 120 ±7 and Day 180±7 for knowledge level, symptom control, medication adherence, and quality of life.

Study design

This study will be conducted at the Hospital Raja Permaisuri Bainun in the state of Perak, Malaysia. A pharmacist-led educational protocol that has been validated by the researchers in other study will be adapted as an interventional tool for this study. This pharmacist led-protocol consists of patient education material and structured pharmacist counselling.

This trial is a single-centre, randomised, two-arm, and parallel-group controlled trial that will be conducted to determine the effectiveness of the pharmacist-led educational protocol. Adult patients diagnosed with moderate to severe AR who attend an outpatient ENT (ear, nose and throat) clinic in Hospital Raja Permaisuri Bainun will be recruited and randomly allocated at a 1:1 ratio into control and intervention groups.

The control arm will receive the current standard of pharmaceutical care, i.e., it is a health service that patient obtaining their medication from the outpatient pharmacy following a session of medical consultation at the ENT clinic, whereas the intervention arm receives the educational material and pharmacist counselling plus standard pharmaceutical care.

Baseline data will be collected and both groups will be followed up post-randomization on Day 60 ± 7, Day 120±7 and Day 180±7. Participants will be contacted virtually (Zoom, Google Meet, or Whatapps call, or telephone call) or face-to-face, depending on the feasibility and standard operating procedures of the outpatient ENT (ear, nose, and throat) clinic at Hospital Raja Permaisuri Bainun during the follow-up on Day 60 ± 7 and Day 120±7. Meanwhile, the end-of-study follow up will be done at the routine scheduled clinic visit, which is around Day 180 ±7.

Diagram of study design

The study design of a prospective, randomized, controlled, parallel group trial is shown in Diagram 1.

Diagram 1: Design of a prospective, randomized, controlled, parallel group trial

Study population

Patients who attended ENT outpatient clinic at Hospital Raja Permaisuri Bainun in the period of study that fulfil the study criteria as below:

Inclusion

1. Malaysian, aged 18 to 80, with medical diagnoses of moderate to severe allergic rhinitis graded according to ARIA guidelines, who attended an ENT outpatient clinic in the period of study, and
2. capable of reading and writing in English or Malay

Exclusion Criteria:

1. Pregnant or lactating mothers.
2. Patients who had comorbid diagnosis of chronic rhinosinusitis.
3. Patients who had psychiatric problems or dementia, at the discretion of the physician, were deemed unfit to participate in this study.
4. Patients with terminal illnesses, at the discretion of the physician, are unfit to participate in this study.
5. Patients with comorbidities or diseases, at the discretion of the physician, are unfit to participate in this study.
6. Patients with post-covid conditions with symptoms that continue beyond 3 months after being infected, at the discretion of the physician, are unfit to participate in this study (*UpToDate*, 2022).

Sample size

Prior data indicate that in determining patients' knowledge level with regard to the use of intranasal steroids for AR treatment, the mean score of adults with adequate knowledge about allergic rhinitis treatment among controls is estimated to be 1.39 (SD:0.71), and the estimated true mean score of adequate knowledge for interventional subjects is 1.70 (SD: 0.58) assuming 77 participants in each group (Retinasekharan et al., 2021). By using the G-power calculator, this study will need 67 interventional participants and 67 control participants to be able to reject the null hypothesis that the mean scores of the interventional

and control groups are equal with probability (power) 0.8. The Type I error probability associated with this test of this null hypothesis is 0.05. The calculation is shown below:-

Sample size estimation for the mean difference of knowledge level between groups

Intervention group
Mean score: 1.70
Standard Deviation: 0.58

Control group
Mean score: 1.39
Standard Deviation: 0.71

Pooled standard deviation

$$Pooled\ SD = \sqrt{\frac{(n_1 - 1) \times SD_1^2 + (n_2 - 1) \times SD_2^2}{n_1 + n_2 - 2}}$$

= √ [(77-1) X 0.71^2 + (77-1) X 0.58^2]/ (77+77-2)
= 0.648267

Results of the G-power calculator:
F tests - ANOVA: Repeated measures, between factors
Analysis: A priori: Compute required sample size

Input:	Effect size f	= 0.2005347
	α err prob	= 0.05
	Power (1-β err prob)	= 0.80
	Number of groups	= 2
	Number of measurements	= 3
	Correlation among repeated measures	= 0.5
Output:	Noncentrality parameter λ	= 8.0830473
	Critical F	= 3.9128750
	Numerator df	= 1.0000000
	Denominator df	= 132
	Total sample size	= 134
	Actual power	= 0.8057571

To determine the sample size required for the mean difference of symptom control between the intervention and control group, studies using TNSS as a symptom control evaluation tool, with the study design of a randomised controlled trial, recruiting a study population of intermitent or persistent AR, and possibly in Asian countries, were searched. The information from a randomised controlled trial that investigated the change in TNSS after an intervention vs. a placebo in adults in China with intermittent or persistent AR was used to estimate the sample size (Han et al., 2011). The G-power sample size calculator is used by setting the probability (power) at 0.8, the Type I error probability associated with this test of this null hypothesis at 0.05, the effect size at 0.3850550, and the function of repeated measures,

between factors ANOVA analysis. A sample of 18 participants is required for each group. The calculation is shown below.

Sample size estimation for the mean difference of symptom control between groups

Intervention group
Mean score: 3.991
Standard Deviation: 2.2145
n=181; reference: (Han et al., 2011)

Control group
Mean score: 5.704
Standard Deviation: 2.2341
n=182 reference: (Han et al., 2011)

Pooled Standard Deviation:

$= \sqrt{[(181-1) \text{ X } 2.21452^2 + (182-1) \text{ X } 2.23412^2]/ (181+182-2)}$

= 2.2243

Results of the G-power calculator:

F tests - ANOVA: Repeated measures, between factors
Analysis: A priori: Compute required sample size

Input:	Effect size f	= 0.3850550
	α err prob	= 0.05
	Power (1-β err prob)	= 0.80
	Number of groups	= 2
	Number of measurements	= 4
	Correlation among repeated measures	= 0.5
Output:	Noncentrality parameter λ	= 8.5401995
	Critical F	= 4.1300177
	Numerator df	= 1.0000000
	Denominator df	= 34.0000000
	Total sample size	= 36
	Actual power	= 0.8102902

Therefore, 18 samples are required for each group

The mean of adherence rate to intranasal corticosteroid among control is 76.62 (SD: 2.848386), while the adherence rate for the intervention group is 93.94 (SD: 2.84) (Feng et al., 2017). By using G-power calculator, we will need to study 2 intervention and control groups, respectively, to be able to reject the null hypothesis that the adherence for intervention and control participants is equal with a probability (power) of 0.8. The Type I error probability associated with the test of this null hypothesis is 0.05. The calculation is shown below:-

Intervention group
Mean: 93.94
Standard Deviation: 2.84
n=16; reference: (Feng et al., 2017)

Control group
Mean score: 76.62
Standard Deviation: 2.848386
n=13; reference: (Feng et al., 2017)

Pooled Standard Deviation:

$$Pooled\ SD = \sqrt{\frac{(n_1 - 1) \times SD_1{}^2 + (n_2 - 1) \times SD_2{}^2}{n_1 + n_2 - 2}}$$

Pooled standard deviation
$= \sqrt{[(16\text{-}1) \text{ X } 2.84^2 + (13\text{-}1) \text{ X } 2.848386^2]} / (16+13\text{-}2)$
= 2.843730

Results of the G-power calculator:

F tests - ANOVA: Repeated measures, between factors

**Analysis:**	A priori: Compute required sample size		
**Input:**	Effect size f	=	3.0289576
	α err prob	=	0.05
	Power (1-β err prob)	=	0.80
	Number of groups	=	2
	Number of measurements	=	3
	Corr among rep measures	=	0.5
**Output:**	Noncentrality parameter λ	=	55.0475049
	Critical F	=	18.5128205
	Numerator df	=	1.0000000
	Denominator df	=	2.0000000
	Total sample size	=	4
	Actual power	=	0.9350969

A study mapping the naso-ocular symptom scores to EQ-5D-5L utility values was referred to in order to obtain the input of mean scores and standard deviation to estimate the sample size required to compare patients' QoL between two groups. The EQ-5D-5L utility values in mean and standard deviation were corresponding to the disease severity in AR patients, where the utility values of 1.000 ± 0.000, 0.943 ± 0.085, 0.909 ± 0.095, 0.849 0.142, and 0.767 ± 0.175 were corresponding to none, mild, moderate, severe, and most severe groups (Kumanomidou et al., 2022). In this study, patients with moderate to severe allergic rhinitis will be recruited for control and interventional groups, and the aim of the intervention is to improve the symptoms, if not totally absence of symptoms, at least the mild disease state. Assuming that the control group patients would remain in the disease status of moderate to

severe and the intervention group would achieve mild disease status, the EQ-5D-5L utility values corresponding to moderate (0.909 ± 0.095) and mild (0.943 ± 0.085) were selected to simulate the mean utility score of control and intervention, respectively.

The G-Power sample size calculator is used, and by setting the function of a repeated-measure ANOVA statistical test, the probability (power) at 0.8, the Type I error probability associated with this test of this null hypothesis at 0.05, and the effect size at 0.1885976, the sample size of 70 is calculated for each group as shown in the formulation of the calculation below.

Formula of Pooled Standard Deviation:

$$Pooled\ SD = \sqrt{\frac{(n_1 - 1) \times SD_1^2 + (n_2 - 1) \times SD_2^2}{n_1 + n_2 - 2}}$$

Pooled standard deviation

$= \sqrt{[(238-1) \times 0.095^2 + (238-1) \times 0.085^2] / (238+238-2)}$

= 0.090139

Sasmple size estimated using G-power calculator:

F tests - ANOVA: Repeated measures, between factors

Analysis: A priori: Compute required sample size

**Input:**	Effect size f	=	0.1885976
	α err prob	=	0.05
	Power (1-β err prob)	=	0.80
	Number of groups	=	2
	Number of measurements	=	4
	Corr among rep measures	=	0.5
**Output:**	Noncentrality parameter λ	=	7.9674683
	Critical F	=	3.9097293
	Numerator df	=	1.0000000
	Denominator df	=	138
	Total sample size	=	140
	Actual power	=	0.8003899

Therefore, a sample size of 70 are required for each group.

To estimate the sample size required to compare EQ-5D VAS between two groups, the input for the control group was derived from a study that evaluated general health–related quality of life among allergic rhinitis patients, where the mean and standard deviation score of EQ-5D VAS reported was 72.1 ± 19.0 (Hoehle et al., 2017); whereas the input for the intervention group was derived from a study assessing QoL in the general Malaysian population with a reported mean and standard deviation of EQ-5D VAS at 85.5 ± 12.3, with

the rationale that the intervention group would achieve QoL similar to the general population (Shafie et al., 2019). By using the G power calculator and setting the function of a repeated-measures ANOVA statistical test, the probability (power), the Type I error probability associated with this test of the null hypothesis to 0.05, and the effect size to 0.3244754, the sample size of 25 is calculated for each group as shown in the formulation of the calculation below.

Formula of Pooled Standard Deviation:

$$Pooled\ SD = \sqrt{\frac{(n_1 - 1) \times SD_1{}^2 + (n_2 - 1) \times SD_2{}^2}{n_1 + n_2 - 2}}$$

Pooled standard deviation

$= \sqrt{[(150-1) \times 19.0^2 + (1137-1) \times 12.3^2] / (150+1137-2)}$

= 13.251663

Sasmple size estimated using G-power calculator:

F tests - ANOVA: Repeated measures, between factors

**Analysis:**	A priori: Compute required sample size		
**Input:**	Effect size f	=	0.3244754
	α err prob	=	0.05
	Power (1-β err prob)	=	0.80
	Number of groups	=	2
	Number of measurements	=	4
	Corr among rep measures	=	0.5
**Output:**	Noncentrality parameter λ	=	8.4227428
	Critical F	=	4.0426521
	Numerator df	=	1.0000000
	Denominator df	=	48.0000000
	Total sample size	=	50
	Actual power	=	0.8115763

Therefore, a sample size of 25 are required for each group.

While there is no reported raw data for variance or partial eta-squared that is required to generate the effect size for determining the within group mean difference in symptom control and quality of life, a rule of thumb is used to determine the magnitude of the effect size. Generally, three different effect sizes can be generated from the different levels of partial eta-squared when using the rule of thumbs. 0.01 is meant for a small treatment effect, 0.06 is for medium, and 0.14 is for large effect size (Cohen, 1988). For this study, we estimate a small effect in order to yield the highest number of samples, and hence 0.01 is selected for partial eta-squared in order to generate an effect size. The G-power sample size calculator is used by setting the probability (power) at 0.8, the Type I error probability associated with this

test of this null hypothesis at 0.05, and the function of repeated measures, an effect size of 0.1005038 within factors ANOVA analysis. The total sample size calculated is 138, with 69 samples required for each group. The sample size calculation is shown below.

Sample size for within-group differences in symptom control and quality of life

Researcher estimate a small treatment effect size by select 0.01 for Partial eta-squared and input into G-power calculator.

Results of the G-power calculator:

F tests - ANOVA: Repeated measures, within factors
Analysis: A priori: Compute required sample size

Input:	Effect size f	= 0.1005038
	α err prob	= 0.05
	Power (1-β err prob)	= 0.80
	Number of groups	= 2
	Number of measurements	= 4
	Corr among rep measures	= 0.5
	Nonsphericity correction ε	= 1
Output:	Noncentrality parameter λ	= 11.1515193
	Critical F	= 2.6267747
	Numerator df	= 3.0000000
	Denominator df	= 408
	Total sample size	= 138
	Actual power	= 0.8056554

Therefore, 69 samples are required for each group

The sample size selected will be 70 participants for each group after considering the highest sample size calculated for each specific objective. By estimating the predicted drop-out rate of 10%, 77 participants for each group will be required for this phase. A total of 154 participants will be recruited for this study.

Study duration

It is estimated to take approximately 6 months for each participant. There will be four encounters with the participants (including the baseline) within six months, and each one is expected to last about 30 minutes.

Sampling technique

A consecutive sampling method will be applied to recruit adult patients who fulfil the inclusion and exclusion criteria upon their visit to the outpatient ENT clinic. When a patient meets the enrolment criteria and agreed for participation, the designated personnel will randomize the subject. Subjects will be randomized using blocked randomization to

randomly assign the participants into control and intervention groups. An excel sheet will be used to generate a block size of 10 random numbers, 5 in each group. Each number will be kept in opaque, sealed envelopes. Consented participants will be instructed to select the sealed envelopes, and the envelopes will be opened by the researchers. The information will be recorded in the screening and enrolment log (appendix C). Subjects will be randomly assigned in a 1:1 ratio to one of two arms: (1) Intervention, or (2) Control. Measure taking to minimise bias

Instrument

Demography and assessment form

The participants will be asked for their demographic information (date of birth, gender, occupation, ethnicity, and smoking status) and this will be recorded at enrolment. In order to characterise smoking status, the definitions of "current," "former," and "never smoker" were adopted (Centers for Disease Control and Prevention, 2017). Subsequently, participants will be assessed for their medical and allergic history, and information regarding underlying diseases will be recorded at enrolment. This information will be obtained by interview or from patient’s medical record by the researcher. Subjective measurement of severity will be graded according to ARIA guidelines at baseline and during follow-ups on day 60±7, 120±7 and 180±7. Objective measurement of disease severity will be determined via endoscopic assessment for middle turbinate oedema grading by the physician at baseline and end of study (Day 180±7). The endoscopic assessment is one of the routine tasks that physicians perform to determine the disease progression of AR. The rationale that this assessment be performed at baseline and end of study is based on expert opinions where improvement of the middle turbinate can be seen between 4 and 6 months when patients are adhering to the intranasal corticosteroid treatment. To reduce the burden on the physicians and patients, it is deemed appropriate to schedule patients to come for a clinic visit for this assessment alongside seeking medical consultation.

Their concomitant medications, if any, will be gauged in that all types of medicine that the patient is currently taking or has taken in the last 30 days will be evaluated. This includes all prescribed and over-the-counter medications. The medication dose, route, unit, frequency of administration, indication for administration, and dates of medication will be captured. Concomitant medication of study-specific interest which will be recorded during the study period includes antihistamines, corticosteroids, anticholinergics, leukotriene receptor

antagonists, nasal saline, decongestants, and beta-blockers. This information will be recorded in the demography and assessment form (appendix D).

Measurement knowledge of intranasal corticosteroid

A self-administered questionnaire evaluating the knowledge of the participants with regards to their understanding of intranasal corticosteroids is adapted. Permission to adapt this questionnaire as well as to perform a translation into Malay language via the process of linguistic validation methodology has been obtained from the originator (copyright holder). The original questionnaire is shown in appendix E (Retinasekharan et al., 2021). The knowledge will be assessed during the baseline data collection, Day 120±7, and on Day 180 ±7.

Measurement of symptoms control

Total nasal symptom scores (TNSS) (Appendix F), a validated tool that enables patients to self-rate their specific nasal symptoms including nasal obstruction, itching, sneezing, secretion, runny nose, and sleep difficulty on a 4-point Likert scale ranging from 0 (no symptom) to 3 (severe symptom) in the past 12 hours and 2 weeks, is found appropriate to be adapted for symptom control assessment. The psychometric properties of TNSS is belonged to the copyright holder and thus it cannot be shared (Downie et al., 2004). The decision to select this tool is based on careful discussion with the expert opinion (rhinologists) in the consideration of the aspects of its simplicity for administration to the patients, its ability to provide objective measurement of patients' self-reported symptoms, and its potential to be used in daily clinical practice. In addition, TNSS has been widely used in randomised controlled trials and adapted in many countries. It is the most accepted primary efficacy variable that is rated for drug approval in AR by the U.S. Food & Drug Administration (U.S. Food and Drug Administration, 2020). Permission to adapt TNSS to be used in this study has been granted. In addition, permission to translate TNSS into a Malay version has also been granted by following linguistic validation methodology where it involves forward and backward translation, review by clinicians, conduct of cognitive interviews, and proof-reading (Acquadro et al., 2012). Symptom control will be assessed at baseline, on Day 60 ±7, Day 120 ±7, and Day 180 ±7.

Measurement of medication adherence

A self-developed diary card to record participants' medication adherence levels on their daily use of intranasal corticosteroid and if additional sprays are required (appendix G). The use of diaries that require participants to self-report their adherence is deemed more appropriate as it allows a more precise estimation of the number of days that the participant declares they are taking the medication. The diary card will be pre-tested prior to use. This variable will be assessed on Day 60 ±7, Day 120 ±7, and Day 180 ±7.

Measurement of quality of life

The EuroQoL 5-Dimensions, 5-Levels (EQ-5D-5L) (appendix H), is a validated tool that has fulfilled these considerations. This tool has also been adapted by several allergic rhinitis-related studies and is therefore deemed appropriate to be adapted for this study (Hwang et al., 2019; Kumanomidou et al., 2022). The EQ-5D-5L consists of a descriptive system and the EQ VAS. The descriptive system is comprised of five dimensions (Mobility, Self-Care, Usual Activities, Pain/Discomfort, and Anxiety/Depression), with each dimension consisting of five response levels, namely, no difficulties, slight problems, moderate problems, severe problems, and unable to/extreme problems. Respondents indicate their health by marking the appropriate box for each question. The EQ VAS captures the respondent's total present health on a vertical visual analogue scale labelled "The best health you can imagine" and "The worst health you can imagine." EQ VAS measures a patient's overall health perception (EuroQol Research Foundation, 2019; Herdman et al., 2011; Shafie et al., 2019) at baseline, on Day 60 ±7, Day 120 ±7, and Day 180 ±7.

Study variables

Table 2 lists the study variables, their descriptions, and the types of variables for the phase-one study.

Table 2: Study variables

Variables	Description	Types of variables
**Demography of patients**		
Age	Age of the participants	Continuous
Gender	The gender of the participants	Categorical
Highest education level	The highest education level of the participants	Categorical
Occupation	The occupation of the participants will be classified according to the social-economic classification (Office for National Statistics, 2010).	Categorical

Smoking status	• Current smoker: ≥ 100 cigarettes and still smoked daily or quit within 1 year of admission<br>• Former smoker is defined as a former smoker smoked ≥ 100 cigarettes and quit more than 1 year.<br>• Never smoke: an adult who has never smoked, or who has smoked less than 100 cigarettes in his or her lifetime.	Categorical
Comorbidity	Comorbids other than asthma	Categorical
Asthma	The control of asthma for patients who have concurrent asthma.	Categorical
Frequency of AR graded according to ARIA	Intermittent or persistent AR symptoms	Categorical
Severity of AR graded according to ARIA	Mild or moderate/severe AR	Categorical
Endoscopy assessment by physician	Middle turbinate edema grading into normal, focal, multifocal, diffuse, or polypoid edema.	Categorical
Family history of AR	The patient reported an AR family history.	Categorical
Self-reported triggering allergen (if known)	The patient self-reported known allergen	Categorical
Concomitant Medication of Interest	Medication for treating AR	Categorical
**Knowledge of nasal steroid spray**		
Four structured questions	The choice of responses is either yes, not sure, or no	Categorical
**Total Nasal Symptom Score (TNSS)**		
Total Nasal Symptom Score	The sum of the scores of the four nasal symptoms self-rated by the patients will be presented as mean and standard deviation	Continuous
**Medication adherence**	The mean adherence rate will be calculated by dividing the number of doses administered with intranasal steroid spray by the total number of doses the intranasal steroid spray is prescribed for. The rate will be presented in mean scores and standard deviation	Continuous
**EQ-5D-5L**		
Utility value	A utlity score will be generated in the form mean and standard deviation	Continuous
Visual analogue scale (VAS)	VAS will be generated in the form of mean and standard deviation	Continuous

Assessment of efficacy

Data collection

The ENT physicians will screen for potential eligible patients in the ENT clinic and refer them to the researchers. Informed consent must be obtained prior to the participants entering the study and before any protocol-directed procedures are performed. A unique subject identification number (subject ID) will be assigned to each participant; this subject ID will be used throughout the study.

The participants will be asked for their demographic information (date of birth, gender, occupation, ethnicity, and smoking status) and this will be recorded at enrolment. In order to characterise smoking status, the definitions of "current," "former," and "never smoker" were adopted (Centers for Disease Control and Prevention, 2017). Subsequently, participants will be assessed for their medical and allergic history, and information regarding underlying diseases will be recorded at enrolment. This information will be obtained by interview or from medical records. Subjective measurement of allergic rhinitis classification will be graded according to ARIA guidelines at baseline and during follow-ups on day 60±7, 120±7 and 180±7. According to the ARIA guideline, the patients will be asked for their frequency of symptoms experienced, in which intermittent is defined as < 4 days per week or < 4 weeks at a time, while persistent is defined as ≥ 4 days per week and ≥ 4 weeks at a time. Their severity will be rated either as "mild" as defined as normal sleep, daily activities, work/school and no troublesome symptoms; or "moderate/severe" as defined as one or more of the symptoms, including abnormal sleep, impairment of daily activities, sport, leisure, problems at work or school, and troublesome symptoms. The possible allergic classifications will be intermittent mild, intermittent moderate/severe, persistent mild, or persistent moderate/severe.

Meanwhile, objective measurement of disease severity will be determined via endoscopic assessment for middle turbinate oedema grading by the physician at baseline and on Day 180±7. The endoscopic assessment is one of the routine tasks that physicians perform to determine the disease progression of AR. The rationale that this assessment be performed at baseline and end of study is based on expert opinions where improvement of the middle turbinate can be seen between 4 and 6 months when patients are adhering to the intranasal corticosteroid treatment. To reduce the burden on the physicians and patients, it is deemed appropriate to schedule patients to come for a clinic visit for this assessment alongside seeking medical consultation.

Their concomitant medications, if any, will be gauged in that all types of medicine that the patient is currently taking or has taken in the last 30 days will be evaluated. This includes all prescribed and over-the-counter medications. The medication dose, route, unit, frequency of administration, indication for administration, and dates of medication will be captured. Concomitant medication of study-specific interest which will be recorded during the study period includes antihistamines, corticosteroids, anticholinergics, leukotriene receptor antagonists, nasal saline, decongestants, and beta-blockers. This information will be recorded in the demography and assessment form (appendix I).

Face-to-face data collection during their physical visit to the ENT clinic will be performed at baseline and end of study follow-up (Day 180±7). The post randomization follow-ups on 60±7 and day 120±7 will be conducted virtually (Zoom, Google Meet, Whatapps call or telephone call) or face-to-face depending on the feasibility and standard operating procedures of the outpatient ENT clinic at Hospital Raja Permaisuri Bainun during the data collection period. The reason for giving an option of virtual follow-up is to avoid adding a burden to the already extensive ENT clinic, which expects a patient load of 80–160 patients with allergic rhinitis every month. However, if the participant's clinic appointment falls on either day 60±7 or day 120±7, face-to-face data collection will be performed. A range of plus or minus 7 days is allowed for each scheduled time point. The data that will be collected on 60±7 and day 120±7 include disease severity assessment, symptom control assessment, medication adherence, and quality of life.

A checklist is created to ensure that the element of the algorithm of pharmacist management is implemented on participants assigned to the intervention group (Appendix I). Pharmacist-led educational protocol that consists of education material and structured pharmacist counselling. The educational material will be introduced to the participants assigned to the interventional group at baseline while the structured pharmacist counselling will be given to the participant in the interventional group at baseline and on 60±7, 120±7 and 180±7. Table 3 shows a schedule of study procedures.

Table 3: Study schedule

**Timepoint**	**Screening, D0**	**Baseline, D0**	**D60±7**	**D120±7**	**D180±7**
**ENROLLMENT:**					
Eligibility screen	×				

Informed consent		×			
Randomization		×			
Demography & medical history		×			
**INTERVENTION:**					
Educational materials		×			
Pharmacist counselling		×	×	×	×
**ASSESSMENT:**					
Disease severity classification using ARIA guideline		×	×	×	×
Endoscopy assessment		×			×
Knowledge level		×		×	×
Symptoms control		×	×	×	×
Medication adherence*			×	×	×
Quality of life		×	×	×	×
Concomitant medication		×	×	×	×

* Patients will fill-up diary card throughout the study period on medication they have taken

Participants' knowledge levels will be evaluated by self-completing a questionnaire assessing their knowledge (appendix E). Their knowledge level will be evaluated at Day 0 (baseline), Day 120±7, and Day 180±7 during their clinic visit.

Participants' symptom control will be assessed by the researcher using TNSS (appendix F). They will be asked to reflect on their symptoms in the past 12 hours and the past 2 weeks. Symptom control will be monitored at Day 0 (baseline), Day 60 ±7, Day 120±7 and Day 180±7.

Participants' medication adherence levels will be evaluated by asking the subject to record the diary card with details on the prescribed intranasal corticosteroid during the study (appendix G). A nasal spray administration diary card will be given to the participants on Day 0. Their adherence will be assessed during Day 60 ±7, Day 120±7 and Day 180±7. If virtual follow-up is conducted on Day 60±7 and Day 120±7, participants will be asked to take a photo of their diary card and send it to the researchers, while those without a smart phone device will be required to bring along the diary card at the end of follow-up. A phone call will

be made prior to the scheduled visit (Day 180 ±7) to remind them to bring along the diary card. If the participants have a scheduled visit at the clinic, they will be asked to bring along the diary card.

The participants' QoL will be evaluated by self-completing EQ-5D-5L (appendix H). Their QoL will be monitored at Day 0 (baseline), Day 60±7, Day 120±7 and Day 180±7. Two sets of paper EQ-5D-5L will be given to participants without a smart phone for them to self-complete at home during virtual follow-up on Day 60±7 and Day 120±7, and they are required to bring them along to the clinic (Day 180±7). Participants with a smart phone will be asked to complete the questionnaire electronically (Google Form) during virtual follow-up on Day 60±7 and Day 120±7. If their follow-up appointment is at the same time as a scheduled clinic visit, they can complete the questionnaire on paper or online (using a Google form).

Statistical analysis

An intention-to-treat analysis will be used to include all of the patients who were assigned to each group after randomization. The data analysis will be done using SPSS version 20.0. Descriptive data will be expressed as mean and standard deviation (SD) if the data is normally distributed, or median and interquartile range (IQR) if the data is not normally distributed. Categorical data will be presented in frequency and percentage.

To determine the knowledge level, The “yes” answer will be given a score of two, the “no” answer will be given a zero score, and “not sure” will be given a score of one (Retinasekharan et al., 2021). The outcome will be presented in mean score and standard deviation for each group. Each question will be analysed separately. The knowledge levels will be assessed at baseline, Day 120±7 and on Day 180 ±7. Between-group comparisons will be analysed using Two-way repeated measure ANOVA to compare mean score different between two groups. Meanwhile, the two-way repeated measures ANOVA will show the findings for the within-group comparison. A P-value of < 0.05 will be considered statistically significant.

The Total Nasal Symptom Score (TNSS) is the sum of scores for each of nasal congestion, sneezing, nasal itching, and rhinorrhoea at each time point, using a four-point scale (0–3), where 0 indicates no symptoms, a score of 1 for mild symptoms that are easily tolerated, a

score of 2 for awareness of symptoms that are bothersome but tolerable, and a score of 3 is reserved for severe symptoms that are hard to tolerate and interfere with daily activity. A higher score indicates the more severe the symptoms are, and the main outcome of TNSS will be generated as a mean and standard deviation. A mean score will be calculated separately for the past 12 hours and past 2 week. A two-way repeated measure ANOVA analysis will be employed to determine the mean difference between the scores of the control group and the interventional group in order to determine the overall effectiveness of the intervention. Within-group comparisons will be shown as part of the outcome of the two-way repeated measures ANOVA. A value of $P < 0.05$ is considered statistically.

In the determination of self-reported medication adherence, a two-way repeated measure ANOVA analysis will be used to compare the between-group differences for the mean adherence rate of number of doses of intranasal corticosteroid. It will be calculated as number of doses taken by the participants divided by the total number of doses prescribed that should be taken on regular basis. Meanwhile, the two-way repeated measures ANOVA will show the findings for the within-group comparison. A value of $P < 0.05$ is considered statistically significant.

The participants' quality of life will be presented as a mean score and standard deviation for responses in EQ-5D-5L utility index scores. The index score will be generated by using the EQ-5D-5L index calculator for Malaysia upon getting permission from the originator (copyright holder) (Shafie et al., 2019). EQ-5D VAS will be generated in the form of a mean score and standard deviation. A two-way repeated measure ANOVA analysis will be employed to determine the mean difference between the scores of the control group and the interventional group. Within-group findings will be obtained from the analysis of two-way repeated measure ANOVA. A value of $P < 0.05$ is considered statistically significant.

Participant withdrawal & drop-out

The participants are free to withdraw from the study at any time for any reason. For safety reasons, the participants may also be withdrawn from the study at any time at the discretion of the investigator. Reasons for withdrawal will be documented. The participant who withdrew will not be replaced. In the event of pregnancy, the patients will be instructed to inform the investigators and physician if she becomes pregnant during the study. Should the

participants become pregnant, she will be discontinued from the study. Since there is no investigational product is involved, the participants will not be follow up thereafter.

Permitted and not permitted medications / treatments during trial

This study did not involve any new investigational products or new invasive procedures or devices. Participants in the intervention will be exposed to educational materials and structured pharmacist counselling. Participants in both groups will continue to receive the usual care at the ENT outpatient clinic and pharmaceutical service at the outpatient pharmacy. There is no issue pertaining to the permitted or not permitted medications/treatments during this study. Participants are allowed to take all the prescribed medications; the over the counter medication consists of medication of interest (Antihistamine, Corticosteroids, Anticholinergics, Leukotriene receptor antagonists, Nasal saline, Decongestants, Beta- blocker) aimed to treat symptoms of allergic rhinitis that the participant purchased outside will be recorded.

There is no conclusive evidence to prove the use of traditional and complementary products improves allergic rhinitis significantly. The participants are allowed to take their traditional and complementary products as per their own routine.

Rescue medication / procedure.

The intervention group is receiving education materials as well as structured pharmacist counselling, and hence the risk of being exposed to unforeseeable risk is minimal. Should the participants have a flare-up of allergic rhinitis symptoms, they are advised to seek medical attention from the ENT outpatient clinic without waiting for their scheduled clinic appointment. If the patient's allergic rhinitis co-exists with asthma, they will be advised to go to the emergency & trauma department or primary health clinic nearest to them without waiting for their scheduled clinic visit. The participants will be advised to call the principal investigator after they have visited any health institution during out-of-schedule visits. They will be evaluated based on the date of their visit, the health care facility they went to, the medication they were prescribed, and the reason for their visit. They will also be graded for their allergic rhinitis status according to the ARIA guidelines and recorded in the assessment form at out-of-schedule visit at patient’s nearest health instituition (appendix D).

Assessment of safety

While there is no new investigational product or new invasive procedures, or new devices is involved in this study, there is no foreseeable adverse events related to the implementation of this pharmacist-led educational protocol. Should participants in the intervention group experience any side effect of the prescribed medication, it is unlikely due to the interventional approach introduced to them. Nevertheless, the medication side effects experienced by participants will be recorded in the assessment form (Appendix D).

If participants in both groups experience intercurrent illness or they find their symptoms getting worse, they are advised to come to the ENT outpatient clinic in this hospital, emergency & trauma department, or primary health clinic nearest to them without awaiting their scheduled clinic appointment. The participants will be advised to call the principal investigator after they have visited any health institution during out-of-schedule visits. They will be evaluated based on the date of their visit, the health care facility they went to, the medication they were prescribed, and the reason for their visit. They will also be graded for their allergic rhinitis status according to the ARIA guidelines and recorded in the assessment form at out-of-schedule visit at patient’s nearest health instituition (appendix D).

Screening failures

All patients who visited an ENT outpatient clinic during the study period will be screened for eligibility. All patients who are screened will be recorded in the Screening and Enrolment Log, which includes screen failures. This log will be kept in the Investigator's Study File (Appendix C).

Lost to follow up

Subject will be considered lost to follow up if they failed to be contacted without stating to withdraw consent. The researchers must attempt to contact the subject (3 phone calls per day for 3 consecutive days and if necessary, a letter to the subject’s last known mailing address) before being considered as lost to follow up. These contact attempts should be documented in the subject file.

Criteria for terminating a study

This study involved pharmacist-led educational intervention, which consists of patient education materials and structured pharmacist counselling, and is to be implemented among

the participants randomly assigned to the intervention group. Meanwhile, the participants in the control group will continue receiving standard pharmaceutical care. The foreseeable risk to the participants in both groups is minimal. There is no involvement of any new investigational product or new invasive procedures in this study. With the consideration of these factors, there are no unforeseeable circumstantials leading to the termination of this study.

Study timeline

Research Activities	2022	2023		2024
	Jul –Dec	Jan- Jun	Jul- Dec	Jan- Jun
Ethical application and approval from USM and NMRR				
Data collection				
Data analysis and interpretation				
Report writing and publication				

References

Abdullah, B., Kandiah, R., Hassan, N.F.H.N., Ismail, A.F., Mohammad, Z.W., Wang, D.Y., 2020. Assessment of perception, attitude, and practice of primary care practitioners towards allergic rhinitis practice guidelines: Development and validation of a new questionnaire. World Allergy Organization Journal 13, 100482. https://doi.org/10.1016/j.waojou.2020.100482

Acquadro, C., Conway, K., Christelle, G., I, M., 2012. Linguistic Validation Manual for Health Outcome Assessments.

Arsoy, G., Varış, A., Saloumi, L.M., Abdi, A., Başgut, B., 2018. Insights on allergic rhinitis management from a Northern Cyprus perspective and evaluation of the impact of pharmacist-led educational intervention on patients' outcomes. Medicina (Lithuania) 54. https://doi.org/10.3390/medicina54050083

Baldacci, S., Maio, S., Angino, A., Sarno, G., Cerrai, S., Simoni, M., Silvi, P., Di Pede, F., Pala, A.P., Bresciani, M., Viegi, G., 2013. Determinants of non-adherence to pharmacologic allergic rhinitis treatment. European Respiratory Journal 42, P3839.

Bender, B.G., 2015. Motivating Patient Adherence to Allergic Rhinitis Treatments. Current Allergy and Asthma Reports 15. https://doi.org/10.1007/s11882-014-0507-8

Blanc, P., Trupin, L., Eisner, M., Earnest, G., Katz, P., Israel, L., Yelin, E., 2001. The work impact of asthma and rhinitis. Journal of Clinical Epidemiology - J CLIN EPIDEMIOL 54, 610–618. https://doi.org/10.1016/S0895-4356(00)00349-8

Bosnic-Anticevich, S., Costa, E., Menditto, E., Lourenço, O., Novellino, E., Bialek, S., Briedis, V., Buonaiuto, R., Chrystyn, H., Cvetkovski, B., Di Capua, S., Kritikos, V., Mair, A., Orlando, V., Paulino, E., Salimäki, J., Söderlund, R., Tan, R., Williams, D.M., Wroczynski, P., Agache, I., Ansotegui, I.J., Anto, J.M., Bedbrook, A., Bachert, C., Bewick, M., Bindslev-Jensen, C., Brozek, J.L., Canonica, G.W., Cardona, V., Carr, W., Casale, T.B., Chavannes, N.H., Correia de Sousa, J., Cruz, A.A., Czarlewski, W., De Carlo, G., Demoly, P., Devillier, P., Dykewicz, M.S., Gaga, M., El-Gamal, Y., Fonseca, J., Fokkens, W.J., Guzmán, M.A., Haahtela, T., Hellings, P.W., Illario, M., Ivancevich, J.C., Just, J., Kaidashev, I., Khaitov, M., Khaltaev, N., Keil, T., Klimek, L., Kowalski, M.L., Kuna, P., Kvedariene, V., Larenas-Linnemann, D.E., Laune, D., Le, L.T.T., Lodrup Carlsen, K.C., Mahboub, B., Maier, D., Malva, J., Manning, P.J., Morais-Almeida, M., Mösges, R., Mullol, J., Münter, L., Murray, R., Naclerio, R., Namazova-Baranova, L., Nekam, K., Nyembue, T.D., Okubo, K., O'Hehir, R.E., Ohta, K., Okamoto, Y., Onorato, G.L., Palkonen, S., Panzner, P., Papadopoulos, N.G., Park, H.S., Pawankar, R., Pfaar, O., Phillips, J., Plavec, D., Popov, T.A., Potter, P.C., Prokopakis, E.P., Roller-Wirnsberger, R.E., Rottem, M., Ryan, D., Samolinski, B., Sanchez-Borges, M., Schunemann, H.J., Sheikh, A., Sisul, J.C., Somekh, D., Stellato, C., To, T., Todo-Bom, A.M., Tomazic, P.V., Toppila-Salmi, S., Valero, A., Valiulis, A., Valovirta, E., Ventura, M.T., Wagenmann, M., Wallace, D., Waserman, S., Wickman, M., Yiallouros, P.K., Yorgancioglu, A., Yusuf, O.M., Zar, H.J., Zernotti, M.E., Zhang, L., Zidarn, M., Zuberbier, T., Bousquet, J., 2019. ARIA pharmacy 2018 "Allergic rhinitis care pathways for community pharmacy": AIRWAYS ICPs initiative (European Innovation Partnership on Active and Healthy Ageing, DG CONNECT and DG Santé) POLLAR (Impact of Air POLLution on Asthma and Rhinitis) GARD Demo. Allergy: European Journal of Allergy and Clinical Immunology 74, 1219–1236. https://doi.org/10.1111/all.13701

Bousquet, J., Schünemann, H.J., Togias, A., Bachert, C., Erhola, M., Hellings, P.W., Klimek, L., Pfaar, O., Wallace, D., Ansotegui, I., Agache, I., Bedbrook, A., Bergmann, K.C., Bewick, M., Bonniaud, P., Bosnic-Anticevich, S., Bossé, I., Bouchard, J., Boulet, L.P., Brozek, J., Brusselle, G., Calderon,

M.A., Canonica, W.G., Caraballo, L., Cardona, V., Casale, T., Cecchi, L., Chu, D.K., Costa, E.M., Cruz, A.A., Czarlewski, W., D'Amato, G., Devillier, P., Dykewicz, M., Ebisawa, M., Fauquert, J.L., Fokkens, W.J., Fonseca, J.A., Fontaine, J.F., Gemicioglu, B., van Wijk, R.G., Haahtela, T., Halken, S., Ierodiakonou, D., Iinuma, T., Ivancevich, J.C., Jutel, M., Kaidashev, I., Khaitov, M., Kalayci, O., Kleine Tebbe, J., Kowalski, M.L., Kuna, P., Kvedariene, V., La Grutta, S., Larenas-Linnemann, D., Lau, S., Laune, D., Le, L., Lieberman, P., Lodrup Carlsen, K.C., Lourenço, O., Marien, G., Carreiro-Martins, P., Melén, E., Menditto, E., Neffen, H., Mercier, G., Mosgues, R., Mullol, J., Muraro, A., Namazova, L., Novellino, E., O'Hehir, R., Okamoto, Y., Ohta, K., Park, H.S., Panzner, P., Passalacqua, G., Pham-Thi, N., Price, D., Roberts, G., Roche, N., Rolland, C., Rosario, N., Ryan, D., Samolinski, B., Sanchez-Borges, M., Scadding, G.K., Shamji, M.H., Sheikh, A., Bom, A.M.T., Toppila-Salmi, S., Tsiligianni, I., Valentin-Rostan, M., Valiulis, A., Valovirta, E., Ventura, M.T., Walker, S., Waserman, S., Yorgancioglu, A., Zuberbier, T., 2020. Next-generation Allergic Rhinitis and Its Impact on Asthma (ARIA) guidelines for allergic rhinitis based on Grading of Recommendations Assessment, Development and Evaluation (GRADE) and real-world evidence. Journal of Allergy and Clinical Immunology 145, 70-80.e3. https://doi.org/10.1016/j.jaci.2019.06.049

Bousquet, J., van Cauwenberge, P., Khaltaev, N., 2004. ARIA in the pharmacy: management of allergic rhinitis symptoms in the pharmacy. Allergic Rhinitis and its Impact on Asthma. Allergy 59, 373–387. https://doi.org/10.1111/j.1398-9995.2003.00468.x

Bousquet, J., van Cauwenberge, P., Khaltaev, N., 2001. Allergic Rhinitis and Its Impact on Asthma. Journal of Allergy and Clinical Immunology 108, S147–S334. https://doi.org/10.1067/mai.2001.118891

Bousquet, J.J., Schünemann, H.J., Togias, A., Erhola, M., Hellings, P.W., Zuberbier, T., Agache, I., Ansotegui, I.J., Anto, J.M., Bachert, C., Becker, S., Bedolla-Barajas, M., Bewick, M., Bosnic-Anticevich, S., Bosse, I., Boulet, L.P., Bourrez, J.M., Brusselle, G., Chavannes, N., Costa, E., Cruz, A.A., Czarlewski, W., Fokkens, W.J., Fonseca, J.A., Gaga, M., Haahtela, T., Illario, M., Klimek, L., Kuna, P., Kvedariene, V., Le, L.T.T., Larenas-Linnemann, D., Laune, D., Lourenço, O.M., Menditto, E., Mullol, J., Okamoto, Y., Papadopoulos, N., Pham-Thi, N., Picard, R., Pinnock, H., Roche, N., Roller-Wirnsberger, R.E., Rolland, C., Samolinski, B., Sheikh, A., Toppila-Salmi, S., Tsiligianni, I., Valiulis, A., Valovirta, E., Vasankari, T., Ventura, M.T., Walker, S., Williams, S., Akdis, C.A., Annesi-Maesano, I., Arnavielhe, S., Basagana, X., Bateman, E., Bedbrook, A., Bennoor, K.S., Benveniste, S., Bergmann, K.C., Bialek, S., Billo, N., Bindslev-Jensen, C., Bjermer, L., Blain, H., Bonini, M., Bonniaud, P., Bouchard, J., Briedis, V., Brightling, C.E., Brozek, J., Buhl, R., Buonaiuto, R., Canonica, G.W., Cardona, V., Carriazo, A.M., Carr, W., Cartier, C., Casale, T., Cecchi, L., Cepeda Sarabia, A.M., Chkhartishvili, E., Chu, D.K., Cingi, C., Colgan, E., De Sousa, J.C., Courbis, A.L., Custovic, A., Cvetkosvki, B., Damato, G., Da Silva, J., Dantas, C., Dokic, D., Dauvilliers, Y., Dedeu, A., De Feo, G., Devillier, P., Di Capua, S., Dykewickz, M., Dubakiene, R., Ebisawa, M., El-Gamal, Y., Eller, E., Emuzyte, R., Farrell, J., Fink-Wagner, A., Fiocchi, A., Fontaine, J.F., Gemicioğlu, B., Schmid-Grendelmeir, P., Gamkrelidze, A., Garcia-Aymerich, J., Gomez, M., Diaz, S.G., Gotua, M., Guldemond, N.A., Guzmán, M.A., Hajjam, J., O'Hourihane, J.B., Humbert, M., Iaccarino, G., Ierodiakonou, D., Ivancevich, J.C., Joos, G., Jung, K.S., Jutel, M., Kaidashev, I., Kalayci, O., Kardas, P., Keil, T., Khaitov, M., Khaltaev, N., Kleine-Tebbe, J., Kowalski, M.L., Kritikos, V., Kull, I., Leonardini, L., Lieberman, P., Lipworth, B., Lodrup Carlsen, K.C., Loureiro, C.C., Louis, R., Mair, A., Marien, G., Mahboub, B., Malva, J., Manning, P., De Manuel Keenoy, E., Marshall, G.D., Masjedi, M.R., Maspero, J.F., Mathieu-Dupas, E., Matricardi, P.M., Melén, E., Melo-Gomes, E., Meltzer, E.O., Mercier, J., Miculinic, N., Mihaltan, F., Milenkovic, B., Moda, G., Mogica-Martinez, M.D.,

Mohammad, Y., Montefort, S., Monti, R., Morais-Almeida, M., Mösges, R., Münter, L., Muraro, A., Murray, R., Naclerio, R., Napoli, L., Namazova-Baranova, L., Neffen, H., Nekam, K., Neou, A., Novellino, E., Nyembue, D., O'Hehir, R., Ohta, K., Okubo, K., Onorato, G., Ouedraogo, S., Pali-Schöll, I., Palkonen, S., Panzner, P., Park, H.S., Pépin, J.L., Pereira, A.M., Pfaar, O., Paulino, E., Phillips, J., Plavec, D., Popov, T.A., Portejoie, F., Price, D., Prokopakis, E.P., Pugin, B., Raciborski, F., Rajabian-Söderlund, R., Reitsma, S., Rodo, X., Romano, A., Rosario, N., Rottem, M., Ryan, D., Salimäki, J., Sanchez-Borges, M.M., Sisul, J.C., Solé, D., Somekh, D., Sooronbaev, T., Sova, M., Spranger, O., Stellato, C., Stelmach, R., Ulrik, C.S., Thibaudon, M., To, T., Todo-Bom, A., Tomazic, P. V., Valero, A.A., Valenta, R., Valentin-Rostan, M., Van Der Kleij, R., Vandenplas, O., Vezzani, G., Viart, F., Viegi, G., Wallace, D., Wagenmann, M., Wang, D.Y., Waserman, S., Wickman, M., Williams, D.M., Wong, G., Wroczynski, P., Yiallouros, P.K., Yorgancioglu, A., Yusuf, O.M., Zar, H.J., Zeng, S., Zernotti, M., Zhang, L., Zhong, N.S., Zidarn, M., 2019. Next-generation ARIA care pathways for rhinitis and asthma: A model for multimorbid chronic diseases. Clinical and Translational Allergy 9, 1–15. https://doi.org/10.1186/s13601-019-0279-2

Bridgeman, M.B., 2017. Overcoming barriers to intranasal corticosteroid use in patients with uncontrolled allergic rhinitis. IPRP Volume 6, 109–119. https://doi.org/10.2147/IPRP.S129544

Canonica, G.W., Triggiani, M., Senna, G.E., 2015. 360 degree perspective on allergic rhinitis management in Italy: A survey of GPs, pharmacists and patients. Clinical and Molecular Allergy 13, 1–8. https://doi.org/10.1186/s12948-015-0029-5

Centers for Disease Control and Prevention, 2017. Adult tobacco use information: glossary [WWW Document]. U.S. Department of Health & Human Services. URL https://www.cdc.gov/nchs/nhis/tobacco/tobacco_glossary.htm (accessed 3.31.22).

Chisholm-Burns, M.A., Spivey, C.A., 2012. The "cost" of medication nonadherence: consequences we cannot afford to accept. J Am Pharm Assoc (2003) 52, 823–826. https://doi.org/10.1331/JAPhA.2012.11088

Chong, S.N., Chew, F.T., 2018. Epidemiology of allergic rhinitis and associated risk factors in Asia. World Allergy Organization Journal 11. https://doi.org/10.1186/s40413-018-0198-z

Cohen, J., 1988. Statistical Power Analysis for the Behavioral Sciences, 2nd ed. Routledge, New York.

Cvetkovski, B., Cheong, L., Tan, R., Kritikos, V., Rimmer, J., Bousquet, J., Yan, K., Bosnic-Anticevich, S., 2020. Qualitative Exploration of Pharmacists' Feedback Following the Implementation of an "Allergic Rhinitis Clinical Management Pathway (AR-CMaP)" in Australian Community Pharmacies. Pharmacy 8, 90. https://doi.org/10.3390/pharmacy8020090

Cvetkovski, B., Kritikos, V., Tan, R., Yan, K., Azzi, E., Srour, P., Bosnic-Anticevich, S., 2019. A qualitative investigation of the allergic rhinitis network from the perspective of the patient. npj Prim. Care Respir. Med. 29, 35. https://doi.org/10.1038/s41533-019-0147-5

Cvetkovski, B., Tan, R., Kritikos, V., Yan, K., Azzi, E., Srour, P., Bosnic-Anticevich, S., 2018. A patient-centric analysis to identify key influences in allergic rhinitis management. npj Primary Care Respiratory Medicine 28, 1–8. https://doi.org/10.1038/s41533-018-0100-z

Dierick, B.J.H., van der Molen, T., Flokstra-de Blok, B.M.J., Muraro, A., Postma, M.J., Kocks, J.W.H., van Boven, J.F.M., 2020. Burden and socioeconomics of asthma, allergic rhinitis, atopic dermatitis and food allergy. Expert Review of Pharmacoeconomics and Outcomes Research 20, 437–453. https://doi.org/10.1080/14737167.2020.1819793

Downie, S.R., Andersson, M., Rimmer, J., Leuppi, J.D., Xuan, W., Akerlund, A., Peat, J.K., Salome, C.M., 2004. Symptoms of persistent allergic rhinitis during a full calendar year in house dust mite-sensitive subjects. Allergy 59, 406–414. https://doi.org/10.1111/j.1398-9995.2003.00420.x

Dupont, W., Plummer, W., 1990. Power and Sample Size Calculations: A Review and Computer Program. Contrlled Clinical Trial 11, 116–28.

EuroQol Research Foundation, 2019. EQ-5D-5L User Guide [WWW Document]. URL https://euroqol.org/publications/user-guides (accessed 6.29.22).

Feng, S., Liang, Z., Zhang, R., Liao, W., Chen, Y., Fan, Y., Li, H., 2017. Effects of mobile phone WeChat services improve adherence to corticosteroid nasal spray treatment for chronic rhinosinusitis after functional endoscopic sinus surgery: a 3-month follow-up study. Eur Arch Otorhinolaryngol 274, 1477–1485. https://doi.org/10.1007/s00405-016-4371-0

Han, D., Liu, S., Zhang, Y., Wang, J., Wang, D., Kong, W., Wang, S., Cheng, L., Zhang, L., The Chinese Allergic Rhinitis Collaborative R, 2011. Efficacy and safety of fluticasone furoate nasal spray in Chinese adult and adolescent subjects with intermittent or persistent allergic rhinitis. allergy asthma proc 32, 472–481. https://doi.org/10.2500/aap.2011.32.3474

Hellings, P.W., Dobbels, F., Denhaerynck, K., Piessens, M., Ceuppens, J.L., De Geest, S., 2012. Explorative study on patient’s perceived knowledge level, expectations, preferences and fear of side effects for treatment for allergic rhinitis. Clinical and Translational Allergy 2, 9. https://doi.org/10.1186/2045-7022-2-9

Herdman, M., Gudex, C., Lloyd, A., Janssen, M., Kind, P., Parkin, D., Bonsel, G., Badia, X., 2011. Development and preliminary testing of the new five-level version of EQ-5D (EQ-5D-5L). Qual Life Res 20, 1727–1736. https://doi.org/10.1007/s11136-011-9903-x

Hoehle, L.P., Speth, M.M., Phillips, K.M., Gaudin, R.A., Caradonna, D.S., Gray, S.T., Sedaghat, A.R., 2017. Association between symptoms of allergic rhinitis with decreased general health-related quality of life. Am J Rhinol Allergy 31, 235–239. https://doi.org/10.2500/ajra.2017.31.4444

Hwang, T.-Y., Kim, S.-K., Kim, S.-H., Kim, M., 2019. A cross sectional survey on health-related quality of life among parents of children with allergic symptoms using the EQ-5D-5L. J Asthma 56, 1239–1245. https://doi.org/10.1080/02770903.2019.1571086

Irwani Ibrahim, Bao Li Chew, Wai Wai Zaw, and H.P.V.B., 2014. The status quo and unmet needs in the management of allergic rhinitis and chronic rhinosinusitis: a Malaysian perspective. Asia Pacific Association of Allergy, Asthma and Clinical Immunology. 4, 164–171.

Jaafa, S., Noh, K.M., Muttalib, K.A., Othman, N.H., Healy, J., 2013. Health Systems in Transition: Malaysia Health System Review.

José, J., Cvetkovski, B., Kritikos, V., Tan, R., Bosnic-Anticevich, S., Lourenço, O., 2020. Interventions Delivered in the Community Pharmacy to Manage Allergic Rhinitis- A Systematic Review of the Literature. Pharmacy 8, 80. https://doi.org/10.3390/pharmacy8020080

Kumanomidou, H., Kanai, K., Oka, A., Haruna, T., Hirata, Y., Makihara, S.-I., Higaki, T., Akamatsu, M., Okamoto, Y., Ikeda, S., Okano, M., 2022. Mapping naso-ocular symptom scores to EQ-5D-5L utility values in Japanese cedar pollinosis. Allergol Int 71, 207–213. https://doi.org/10.1016/j.alit.2021.11.002

Lim, F.L., Hashim, Z., Than, L.T.L., Said, S.M., Hashim, J.H., Norbäck, D., 2015. Asthma, airway symptoms and rhinitis in office workers in Malaysia: Associations with house dust mite (HDM) allergy, cat allergy and levels of house dust mite allergens in office dust. PLoS ONE 10, 1–21. https://doi.org/10.1371/journal.pone.0124905

Lourenço, O., Bosnic-Anticevich, S., Costa, E., Fonseca, J.A., Menditto, E., Cvetkovski, B., Kritikos, V., Tan, R., Bedbrook, A., Scheire, S., Bachert, C., Białek, S., Briedis, V., Boussery, K., Canonica, G.W., Haahtela, T., Kuna, P., Novellino, E., Samoliński, B., Schünemann, H.J., Wallace, D., Bousquet, J., 2020. Managing Allergic Rhinitis in the Pharmacy: An ARIA Guide for Implementation in Practice. Pharmacy 8, 85. https://doi.org/10.3390/pharmacy8020085

Meltzer, E.O., Bukstein, D.A., Hamrah, P.M., Scott, N., Welz, J.A., 2017. Value-Based Perspectives on the Management of Allergic Rhinitis 12.

Office for National Statistics, 2010. Standard occupational classification 2010. (rebased on the SOC2010) user manual Vol. 3, Vol. 3,. Palgrave Macmillan, Basingstoke.

Ozdoganoglu, T., Songu, M., 2012. The burden of allergic rhinitis and asthma. Therapeutic Advances in Respiratory Disease 6, 11–23. https://doi.org/10.1177/1753465811431975

Retinasekharan, S., Md Shukri, N., Ismail, A.F., Abdullah, B., 2021. Knowledge, Attitude, and Practice of Intranasal Corticosteroid in Allergic Rhinitis Patients: Development of a New Questionnaire. Healthcare (Basel, Switzerland) 10. https://doi.org/10.3390/healthcare10010008

Salkind, N., 2022. Encyclopedia of Research Design. https://doi.org/10.4135/9781412961288

Shafie, A.A., Vasan Thakumar, A., Lim, C.J., Luo, N., 2019. Psychometric performance assessment of Malay and Malaysian English version of EQ-5D-5L in the Malaysian population. Qual Life Res 28, 153–162. https://doi.org/10.1007/s11136-018-2027-9

Simons, F.E.R., 1996. Learning Impairment and Allergic Rhinitis. allergy asthma proc 17, 185–189. https://doi.org/10.2500/108854196778996895

Smith, S., Porteous, T., Bond, C., Francis, J., Lee, A.J., Lowrie, R., Scotland, G., Sheikh, A., Thomas, M., Wyke, S., Smith, L., 2020. The Help for Hay Fever community pharmacy-based pilot randomised controlled trial for intermittent allergic rhinitis. npj Prim. Care Respir. Med. 30, 23. https://doi.org/10.1038/s41533-020-0180-4

Sullivan, S.D., Weiss, K.B., 2001. Health economics of asthma and rhinitis. II. Assessing the value of interventions. J Allergy Clin Immunol 107, 203–210. https://doi.org/10.1067/mai.2001.112851

Tripathi, A., Patterson, R., 2001. Impact of allergic rhinitis treatment on quality of life. Pharmacoeconomics 19, 891–899. https://doi.org/10.2165/00019053-200119090-00001

UpToDate, 2022. Patient education: Recovery after COVID-19 (The Basics) [WWW Document]. URL https://www.uptodate.com/contents/recovery-after-covid-19-the-basics (accessed 6.23.22).

U.S. Food and Drug Administration, 2020. Allergic Rhinitis: Developing Drug Products for Treatment Guidance for Industry [WWW Document]. U.S. Food and Drug Administration. URL https://www.fda.gov/regulatory-information/search-fda-guidance-documents/allergic-rhinitis-developing-drug-products-treatment-guidance-industry (accessed 6.24.22).

World Allergy Organization (WAO), 2013. Allergic diseases: A global public health issue. World Allergy Organization, Milwaukee, Wisconsin 53202.

Yawn, B.P., 2008. Importance of allergic rhinitis management in achieving asthma control: ARIA update. Expert Review of Respiratory Medicine 2, 713–719. https://doi.org/10.1586/17476348.2.6.713

Appendix A: English version - Participant Information Sheet

PATIENT INFORMATION SHEET AND INFORMED CONSENT FORM

(for adult subjects and interventional studies)

1. **Title of study**: A prospective, randomized, controlled, parallel group, single-centre trial to assess the effectiveness of Pharmacist-led educational protocol in patients with Allergic Rhinitis in a Tertiary Hospital

2. **Name of investigator and institution:**

    i. Chew Chii Chii, Clinical Research Centre, Hospital Raja Permaisuri Bainun, Ipoh, Ministry of Health, Malaysia & School of Pharmaceutical Sciences, University Science of Malaysia
    ii. Dr. Lim Xin Jie Clinical Research Centre, Hospital Raja Permaisuri Bainun, Ipoh, Ministry of Health, Malaysia
    iii. Chan Huan Keat Clinical Research Centre, Hospital Sultanah Bahiyah, Alor Setar, Ministry of Health, Malaysia
    iv. Dr. Doris George Pharmacy Department, Hospital Raja Permaisuri Bainun, Ipoh, Ministry of Health, Malaysia
    v. Dr. Pathma Letchumanan, Department of Otorhinolaryngology, Hospital Raja Permaisuri Bainun, Ipoh, Ministry of Health, Malaysia
    vi. Dr. Philip Rajan Department of Otorhinolaryngology & Clinical Research Centre, Hospital Raja Permaisuri Bainun, Ipoh, Ministry of Health, Malaysia
    vii. Dr. Kelvinder Singh A/L Awtar Singh Department of Otorhinolaryngology, Hospital Raja Permaisuri Bainun, Ipoh, Ministry of Health, Malaysia
    viii. Dr. Loong Siow Ping Department of Otorhinolaryngology, Hospital Raja Permaisuri Bainun, Ipoh, Ministry of Health, Malaysia

3. **Name of sponsor**: no external funding

4. **Introduction:**

You are invited to participate in a research study because you have allergic rhinitis. The details of the research trial are described in this document. It is important that you understand why the research is being done and what it will involve. Please take your time to read through and consider this information carefully before you decide if you are willing to participate. Ask the study staff if anything is unclear or if you'd like more information. After you are properly satisfied that you understand this study and that you

wish to participate, you must sign this informed consent form. To participate in this study, you may be required to provide researchers with information on your health history. You may harm yourself if you are not truthful with the information provided.

Your participation in this study is voluntary. You do not have to be in this study if you do not want to. You may also refuse to answer any questions you do not want to answer. If you volunteer to be in this study, you may withdraw from it at any time. If you withdraw, any data collected from you up to the time of your withdrawal will still be used for the study. Your refusal to participate or withdraw will not affect any medical or health benefits to which you are otherwise entitled.

This study has been approved by the Medical Research and Ethics Committee, Ministry of Health Malaysia.

5. What is the purpose of the study?

The purpose of this study is to determine the effectiveness of a Malaysian pharmacist-led educational protocol for allergic rhinitis management in adult patients. This research is necessary to improve patients' understanding and management of allergic rhinitis.

The aspect of study that is experimental will be the introduction of pharmacist-led educational protocol to the participants who randomly assigned to intervention group while the control will be receiving standard pharmaceutical care.

A total of 154 participants like you visiting this clinic will be participating in this study. The whole study will last about 12 months (from 01/07/2023 to 30/06/2024) and your participation will take about 30 minutes at each encounter. There will be a total of four encounters, including this visit.

6. What kind of procedures will I receive?

If you agree to participate in the study, you will be randomly (by chance, like flipping a coin) assigned to one of the groups below. You have an equal chance of being assigned to each of the groups.

Group 1 will be introducing a pharmacist-led educational interventional protocol including standard pharmaceutical care.

Group 2 will receiving standard pharmaceutical care.

7. What will happen if I decide to take part?

a. Participants randomly assigned to group 1 will receive an education session and structured medication counselling with the pharmacist and then proceed to pharmacy counter to receive standard pharmaceutical care by obtaining their medication as usual.
b. Participants randomly assigned to group 2 will receive standard pharmacy care including medical doctor consultation and to receive standard pharmaceutical care by obtainining their medication at the pharmacy counter.
c. All participants will be followed-up at months 2, 4, and 6 after this initial visit, either by a visit to the ENT clinic or by a virtual meeting (Zoom, Google meeting, or

Whatsapp call or telephone), according to the convenience of the participants. However, all participants are required to come for a clinic visit at month 6 to be assessed by the doctor as a routine check-up.

d. If any of your clinic appointments fall months 2, 4, and 6, you will be given a face-to-face follow-up.

e. At the end of the study, participants in group 2 will receive educational material along with pharmacist counselling.

8. When will I receive the trial product and how should it be kept?

No study product is involved in this study.

9. What are my responsibilities when taking part in this study?

It is important that you answer all of the questions asked by the researcher honestly and completely. If your condition or circumstances change during the study, you must tell the researcher and the medical doctor.

If you feel that your symptoms of allergic rhinitis are not adequately controlled, you must visit the clinic without awaiting your appointment date. Additionally, it is very important that the researcher and medical doctor be informed very rapidly of any eventual changes to your health during your participation in the study. For your own safety, it is important that you follow the medical doctor’s instructions for medication administration.

10. What kind of treatment will I receive after my participation in the trial?

No study product and no additional treatment is involved when you participate in this study. At the end of the study, all participant will be introduced to a pharmacist-led educational protocol for knowledge improvement in allergy rhinitis management.

11. What are the potential risks and side effects of being in this study?

The risk for participants in group 1 is minimal as they will be exposed to educational material and structured counselling by the pharmacists in addition to the standard pharmaceutical care.

The risk for participants in group 2 who received standard pharmaceutical care is minimal.

This study excludes pregnant or breast-feeding mothers. Although there is no trial product involved in this study, if you become pregnant after participating in this study, you must inform your medical doctor in-charge and the researchers. The medical doctor will discuss with the researcher the safety of your continuous participation.

Please ask the researcher if you need more information on risks and side effects. The researcher will inform you in a timely manner about any new findings.

12. What are the benefits of being in this study?

There may or may not be any benefits to you. Information obtained from this study will help improve the treatment or management of other patients with the same disease or condition.

13. What if I am injured during this study?

The risk of participating in this study is minimal. However, if you are injured as a result of participating in this study, you should visit the ENT clinic, Hospital Raja Permaisuri Bainun and consult a medical doctor, or go to the hospital emergency department immediately.

14. What are my alternatives if I do not participate in this study?

You do not have to participate in this study to get treatment for your disease or condition. Your entitlement to medical care and benefits will not be affected.

15. Who is funding the research?

This study does not receive any external funding. You will not be paid for participating in this study.

16. Can the research or my participation be terminated early?

The researcher, after consulting with the medical doctor, may, due to concerns for your safety, stop the study or your participation at any time. If the study is stopped early for any reason, you will be informed and arrangements will be made for your next clinic visit. You may be asked to attend a final follow-up visit.

17. Will my medical information be kept private?

All your information obtained in this study will be kept and handled in a confidential manner in accordance with applicable laws and/or regulations. When publishing or presenting the study results, your identity will not be revealed without your expressed consent. Individuals involved in this study and in your medical care, qualified monitors and auditors, and governmental or regulatory authorities may inspect and copy your medical records, where appropriate and necessary.

18. Will I be informed about the study findings or new information?

You will not be informed about the study findings. The study findings will be disseminated in the form of presentations and publications without revealing the identities of the participants in this study.

You will be informed of new information if it becomes available during the study period.

19. Who should I call if I have questions?

If you have any questions about the study or if you think you have a study related injury and you want information about treatment, please contact the researcher, Chew Chii Chii at telephone number 05-208 5148. If you have any questions about your rights as a participant in this study, please contact: The Secretary, Medical Research & Ethics Committee, Ministry of Health Malaysia, at telephone number 03-3362 8407 / 8205 / 8888.

INFORMED CONSENT FORM

Title of Study: Development of a Malaysian pharmacist-led educational protocol for allergic rhinitis management in adult patients

By signing below I confirm the following:

- I have been given oral and written information for the above study and have read and understood the information given.
- I have had sufficient time to consider participation in the study and have had the opportunity to ask questions and all my questions have been answered satisfactorily.
- I understand that my participation is voluntary and I can at any time free withdraw from the study without giving a reason and this will in no way affect my future treatment. I am not taking part in any other research study at this time. I understand the risks and benefits, and I freely give my informed consent to participate under the conditions stated. I understand that I must follow the researchers' instructions related to my participation in the study.
- I understand that study staff, qualified monitors and auditors, the sponsor or its affiliates, and governmental or regulatory authorities, have direct access to my medical record in order to make sure that the study is conducted correctly and the data are recorded correctly. All personal details will be treated as STRICTLY CONFIDENTIAL
- I will receive a copy of this Participant information/informed consent form signed and dated to bring home.

Subject:

Signature: I/C number:

Name: Date:

Investigator conducting informed consent:

Signature: I/C number:

Name: Date:

Appendix A: Malay version - Participant Information Sheet

RISALAH MAKLUMAT PESERTA DAN

BORANG PERSETUJUAN atau KEIZINAN PESERTA

(untuk subjek dewasa)

1. **Tajuk penyelidikan**: Kajian bersifat prospektif, secara rawak, terkawal, kumpulan selari, satu pusat untuk menilai keberkesanan protokol pendidikan yang dilaksanakan oleh Ahli Farmasi terhadap pesakit mengidapi alahan hidung di sebuah hospital tertiari

   A prospective, randomized, controlled, parallel group, single-centre trial to assess the effectiveness of Pharmacist-led educational protocol in patients with Allergic Rhinitis in a Tertiary Hospital

2. **Nama Institusi and nama penyelidik:**
   i. Chew Chii Chii
   Pusat Penyelidikan Klinikal, Hospital Raja Permaisuri Bainun, Ipoh, Kementerian Kesihatan Malaysia & Pusat Pengajian Sains Farmasi, Universiti Sains Malaysia
   ii. Dr. Lim Xin Jie
   Pusat Penyelidikan Klinikal, Hospital Raja Permaisuri Bainun, Ipoh, Kementerian Kesihatan Malaysia
   iii. Dr. Doris George
   Jabatan Farmasi, Hospital Raja Permaisuri Bainun, Ipoh, Kementerian Kesihatan Malaysia
   iv. Dr. Pathma Letchumanan,
   Jabatan Otorinolaringologi, Hospital Raja Permaisuri Bainun, Ipoh, Kementerian Kesihatan Malaysia
   v. Dr. Philip Rajan
   Jabatan Otorinolaringologi & Pusat Penyelidikan Klinikal, Hospital Raja Permaisuri Bainun, Ipoh, Kementerian Kesihatan Malaysia
   vi. Dr. Kelvinder Singh A/L Awtar Singh
   Jabatan Otorinolaringologi, Hospital Raja Permaisuri Bainun, Ipoh, Kementerian Kesihatan Malaysia
   vii. Dr. Loong Siow Ping
   Jabatan Otorinolaringologi, Hospital Raja Permaisuri Bainun, Ipoh, Kementerian Kesihatan Malaysia

3. **Nama penaja**: tidak menerima penajaan/dana dari pihak luar

4. **Pengenalan:**

   Anda telah dijemput untuk menyertai penyelidikan ini kerana anda mempunyai alahan hidung. Risalah ini menjelaskan hal-hal berkenaan penyelidikan tersebut dengan lebih mendalam dan terperinci. Amat penting anda memahami mengapa penyelidikan ini

dilakukan dan apa yang dilakukan dalam penyelidikan ini. Sila ambil masa yang secukupnya untuk membaca dan mempertimbangkan dengan teliti penerangan yang diberi sebelum anda bersetuju untuk menyertai penyelidikan ini. Jika ada sebarang kemusykilan ataupun maklumat lanjut yang anda ingin tahu, anda boleh bertanya dengan mana-mana kakitangan yang terlibat dalam penyelidikan ini. Setelah anda berpuashati bahawa anda memahami penyelidikan ini, dan anda berminat untuk turut serta, anda dikehendaki untuk menandatangani Borang Persetujuan atau Keizinan Peserta, pada muka surat akhir risalah ini. Untuk menyertai penyelidikan ini, anda perlu memberi penyelidik tentang maklumat sejarah kesihatan ataupun penyakit anda yang lalu; jika anda tidak berterusterang anda mungkin boleh menimbulkan masalah pada diri anda di kemudian hari.

Penyertaan anda dalam penyelidikan ini adalah secara sukarela. Anda tidak perlu menyertai penyelidikan ini jika anda tidak mahu. Anda juga mempunyai hak untuk tidak menjawab mana-mana soalan yang anda tidak mahu jawab. Anda juga boleh menarik diri daripada penyelidikan ini pada bila-bila masa. Jika anda menarik diri, segala maklumat yang telah diperolehi sebelum anda menarik diri tetap akan digunakan dalam penyelidikan ini. Jika anda tidak mahu menyertai ataupun menarik diri dari penyelidikan ini, tindakan anda tidak akan menjejaskan segala hak dan keistimewaan perkhidmatan perubatan dan kesihatan yang selayaknya anda terima.

Penyelidikan ini telah mendapat kelulusan Jawatankuasa Etika dan Penyelidikan Perubatan, Kementerian Kesihatan Malaysia.

5. Apakah tujuan penyelidikan ini dilakukan?

Tujuan kajian ini adalah untuk menentukan keberkesanan protokol pendidikan yang diterajui oleh ahli farmasi Malaysia untuk pengurusan resdung bagi pesakit dewasa. Penyelidikan ini adalah perlu untuk meningkatkan pemahaman dan pengurusan resdung.

Aspek kajian yang bersifat eksperimen adalah pengenalan protokol pendidikan yang diterajui oleh ahli farmasi kepada peserta yang secara rawak diagihkan kepada kumpulan intervensi manakala kumpulan kawalan akan menerima perkhidmatan farmasi secara standard.

Seramai 154 peserta seperti anda yang melawat klinik ini akan mengambil bahagian dalam kajian ini. Keseluruhan kajian akan berlangsung kira-kira 12 bulan (dari 01/07/2023 hingga 30/06/2024) dan penyertaan anda akan mengambil masa kira-kira 30 minit pada setiap pertemuan. Terdapat empat pertemuan, termasuk lawatan ini.

6. Apakah prosedur penyelidikan yang akan saya terima?

Jika anda bersetuju untuk mengambil bahagian dalam kajian, anda akan secara rawak (secara kebetulan, seperti membalikkan syiling) ditugaskan kepada salah satu kumpulan di bawah. Peluang untuk anda dimasukkan ke mana-mana kumpulan rawatan adalah sama.

Kumpulan 1 akan diperkenalkan protokol intervensi pendidikan oleh ahli farmasi termasuk perkhidmatan farmasi secara standard.

Kumpulan 2 akan menerima perkhidmatan farmasi secara standard.

7. Apakah yang terjadi kepada saya sekiranya saya bersetuju untuk menyertai penyelidikan ini?

a. Peserta yang dimasukkan secara rawak ke kumpulan 1 akan menerima sesi pendidikan dan kaunseling ubat secara berstruktur dengan ahli farmasi, dan pergi ke kaunter farmasi untuk mendapatkan perkhidmatan farmasi secara standard dengan mengambil ubat seperti biasa.
b. Peserta yang dimasukkan secara rawak ke kumpulan 2 akan menerima perkhidmatan perubatan seperti biasa yang melibatkan perundingan dari doktor perubatan dan mendapatkan perkhidmatan farmasi secara standard di kaunter farmasi untuk mendapatkan ubat seperti biasa.
c. Semua peserta akan disusuli pada bulan ke-2, ke-4, atau ke-6 selepas perjumpaan pertama ini, sama ada dengan lawatan ke klinik ENT atau melalui mesyuarat maya (Zoom, Google meeting, atau panggilan Whatsapp), mengikut keselesaan peserta. Bagaimanapun, semua peserta dikehendaki datang ke klinik pada bulan 6 untuk pemeriksaan rutin oleh doktor.
d. Jika mana-mana temujanji klinik anda jatuh pada bulan ke-2, ke-4, atau ke-6, anda akan diberi susulan secara bersemuka.
e. Pada akhir kajian, peserta dalam kumpulan 2 akan menerima bahan pendidikan bersama dengan kaunseling yang diberi oleh ahli farmasi.

8. Bilakah saya akan menerima produk penyelidikan dan bagaimana cara menyimpannya?

Kajian ini tidak melibatkan sebarang produk kajian.

9. Apakah tanggungjawab saya sewaktu menyertai penyelidikan ini?

Adalah penting anda menjawab semua soalan yang ditanya oleh penyelidik dengan jujur dan lengkap. Jika keadaan atau keadaan anda berubah semasa kajian, anda mesti memberitahu penyelidik dan doktor perubatan.

Jika anda merasakan simptom resdung anda tidak dikawal secukupnya, anda mesti melawat klinik tanpa menunggu tarikh temujanji anda. Di samping itu, adalah sangat penting bahawa penyelidik dan doktor perubatan dimaklumkan dengan cepat tentang sebarang perubahan pada kesihatan anda semasa anda menyertai kajian ini. Untuk keselamatan anda sendiri, adalah penting anda mengikuti arahan doktor perubatan untuk mengambil ubat.

10. Apakah jenis rawatan yang akan saya terima selepas menyertai penyelidikan ini?

Tiada produk kajian dan tiada rawatan tambahan terlibat apabila anda mengambil bahagian dalam kajian ini. Pada akhir kajian, semua peserta akan diperkenalkan dengan protokol pendidikan yang diterajui oleh ahli farmasi untuk peningkatan pengetahuan untuk mengurus resdung.

11. Apakah risiko dan kesan-kesan sampingan menyertai penyelidikan ini?

Risiko untuk peserta dalam kumpulan 1 adalah minimum kerana mereka akan didedahkan kepada bahan pendidikan dan kaunseling bersturktur oleh ahli farmasi selain penerimaan perkhidmatan farmasi seperti biasa

Risiko untuk peserta dalam kumpulan 2 yang menerima perkhidmatan farmasi seperti biasa adalah minimum.

Kajian ini tidak termasuk ibu mengandung atau menyusu. Walaupun tiada produk percubaan yang terlibat dalam kajian ini, jika anda hamil selepas mengambil bahagian dalam kajian ini, anda mesti memaklumkan kepada doktor perubatan anda yang bertanggungjawab dan penyelidik. Doktor perubatan akan membincangkan dengan penyelidik keselamatan penyertaan berterusan anda.

Sila tanya penyelidik jika anda memerlukan maklumat lanjut tentang risiko dan kesan sampingan. Penyelidik akan memaklumkan anda tepat pada masanya tentang sebarang penemuan baharu.

12. Apakah manfaatnya saya menyertai kajian ini?

Penyelidikan ini mungkin akan mendatangkan manfaat ataupun langsung tiada memberi apa- apa manfaat kepada anda. Segala maklumat yang diperolehi daripada penyelidikan ini akan dapat membantu dalam penambahbaikan kaedah rawatan atau pengurusan pesakit lain yang menghidap penyakit atau masalah kesihatan yang sama dengan anda.

13. Apakah yang akan terjadi sekiranya saya tercedera semasa menyertai kajian ini?

Risiko menyertai kajian ini adalah minimum. Walau bagaimanapun, jika anda cedera akibat mengambil bahagian dalam kajian ini, anda harus melawat klinik ENT Hospital Raja Permaisuri Bainun dan berunding dengan doktor perubatan, atau pergi ke jabatan kecemasan hospital dengan segera.

14. Apakah rawatan alternatif lain sekiranya saya tidak menyertai penyelidikan ini?

Anda tidak perlu mengambil bahagian dalam kajian ini untuk mendapatkan rawatan penyakit atau keadaan anda. Kelayakan anda untuk mendapatkan rawatan dan faedah perubatan tidak akan terjejas.

15. Siapakah yang membiayai penyelidikan ini?

Kajian ini tidak menerima sebarang pembiayaan luar. Anda tidak akan dibayar untuk menyertai kajian ini.

16. Bolehkah penyelidikan ataupun penyertaan saya ditamatkan lebih awal daripada yang dirancang?

Penyelidik, selepas berunding dengan doktor perubatan, mungkin, disebabkan kebimbangan terhadap keselamatan anda, menghentikan kajian atau penyertaan anda pada bila-bila masa. Jika kajian dihentikan awal atas sebarang sebab, anda akan

dimaklumkan dan pengaturan akan dibuat untuk untuk lawatan klinik anda yang seterusnya. Anda mungkin diminta untuk menghadiri lawatan susulan terakhir.

17. Adakah maklumat perubatan saya akan dirahsiakan?

Segala maklumat anda yang diperolehi dalam penyelidikan ini akan disimpan dan dikendalikan secara sulit, bersesuaian dengan peraturan-peraturan dan/ atau undang-undang yang berkenaan. Sekiranya hasil penyelidikan ini diterbitkan atau dibentangkan kepada orang ramai, identiti anda tidak akan didedahkan tanpa kebenaran anda terlebih dahulu. Pihak- pihak tertentu seperti individu yang terlibat dalam penyelidikan dan rawatan perubatan anda, juruaudit dan jurupantau yang terlatih, pihak berkuasa kerajaan atau undang-undang, boleh memeriksa dan membuat salinan laporan perubatan anda jika berkenaan dan diperlukan

18. Adakah saya akan dimaklumkan tentang keputusan kajian?

Anda tidak akan dimaklumkan tentang keputusan kajian. Keputusan kajian akan disebarkan dalam bentuk pembentangan dan penerbitan tanpa mendedahkan identiti peserta dalam kajian ini.

Anda akan dimaklumkan tentang maklumat baharu jika ada semasa tempoh kajian.

18. Siapakah yang perlu saya hubungi sekiranya saya mempunyai sebarang pertanyaan?

Anda boleh menghubungi doktor penyelidikan ini Chew Chii Chii pada sambungan telefon 05-208 5148 sekiranya anda mempunyai sebarang pertanyaan mengenai penyelidikan ini atau jika anda mengesyaki anda mengalami kecederaan yang terhasil daripada penyelidikan ini dan anda mahukan maklumat tentang rawatannya.

Jika anda mempunyai sebarang pertanyaan berkaitan dengan hak-hak anda sebagai pesakit dalam penyelidikan ini, sila hubungi: Setiausaha, Jawatankuasa Etika & Penyelidikan Perubatan, Kementerian Kesihatan Malaysia, melalui talian telefon 03-3362 8407/ 8205 / 8888.

BORANG PERSETUJUAN/ KEIZINAN PESAKIT

Tajuk Penyelidikan: Keberkesanan protokol pendidikan yang diterajui ahli farmasi Malaysia untuk pengurusan resdung bagi pesakit dewasa

Dengan menandatangani di bawah, saya mengesahkan bahawa:

- Saya telah diberi maklumat tentang penyelidikan di atas secara lisan dan bertulis and saya telah membaca dan memahami segala maklumat yang diberikan dalam risalah ini.
- Saya telah diberikan masa yang secukupnya untuk mempertimbangkan penyertaan saya dalam penyelidikan ini dan telah diberi peluang untuk bertanyakan soalan dan semua persoalan saya telah dijawab dengan sempurna dan memuaskan.
- Saya juga faham bahawa penyertaan saya adalah secara sukarela dan pada bila-bila masa saya bebas menarik diri daripada penyelidikan ini tanpa harus memberi sebarang alasan dan ianya sama sekali tidak akan menjejaskan rawatan perubatan saya pada masa akan datang. Saya tidak mengambil bahagian dalam mana-mana penyelidikan lain pada masa ini. Saya juga memahami tentang risiko dan manfaat penyelidikan ini dan saya secara sukarela memberi persetujuan untuk menyertai penyelidikan ini di bawah syarat-syarat yang telah dinyatakan di atas. Saya faham saya harus mematuhi nasihat dan arahan yang berkaitan dengan penyertaan saya dalam penyelidikan ini daripada penyelidik.
- Saya faham bahawa kakitangan penyelidikan, pemantau dan juruaudit terlatih , pihak penaja atau gabungannya, dan pihak berkuasa kerajaan atau undang-undang, mempunyai akses langsung dan boleh menyemak laporan perubatan saya bagi memastikan penyelidikan ini dijalankan dengan betul dan data direkodkan dengan betul. Segala maklumat dan data peribadi akan dianggap sebagai SULIT.
- Saya akan menerima satu salinan 'Risalah Maklumat Peserta dan Borang Persetujuan atau Keizinan Peserta' yang telah lengkap dengan tarikh dan tandatangan untuk dibawa pulang ke rumah.

Subjek :

Tandatangan: Nombor K/P:

Nama: Tarikh :

Penyelidik yang mengendalikan proses menandatangani borang keizinan:

Tandatangan: Nombor K/P:

Nama: Tarikh :

Appendix B: Subject Identification Log

**Protocol #:**		**Site Name:**	Hospital Raja Permaisuri Bainun
**Principal Investigator:**		**Site #:**	

Subject Study ID	Hospital RN	Subject Name	Subject Address	Subject Telephone Number

Appendix C: Subject Screening and Enrolment Log

**Site Name:**	

**Subject Study ID**	**Gender**	**Date of Informed Consent** DD-MMM-YYYY	**Screening Date** DD-MMM-YYYY	**Subject Enrolled**	**If Enrolled, insert randomization code**	**Screen Failure/ Completed/ Early Terminated Date**	**Reason for screen failure/ early termination**	**Initial**
	☐ Male<br>☐ Female			☐ Yes<br>☐ No				
	☐ Male<br>☐ Female			☐ Yes<br>☐ No				
	☐ Male<br>☐ Female			☐ Yes<br>☐ No				
	☐ Male<br>☐ Female			☐ Yes<br>☐ No				
	☐ Male<br>☐ Female			☐ Yes<br>☐ No				
	☐ Male<br>☐ Female			☐ Yes<br>☐ No				
	☐ Male<br>☐ Female			☐ Yes<br>☐ No				
	☐ Male<br>☐ Female			☐ Yes<br>☐ No				

**Mark "X" for the appropriate answer*

#Put "NA" if not applicable

Appendix D: Demography and assessment form

Participant demographic

Subject ID				
Age	________________years			
Gender	☐ Male	☐ Female		
Highest education level	Primary	☐ Secondary	☐ Tertiary (diploma and above)	☐ No formal education
Occupation				
Smoking status	☐ Never	☐ Current	☐ Former	
Comorbidity	☐ Dermatitis	☐ Conjunctivitis	☐ Metabolic disease	Other _________
Asthma<br>☐ Not applicable	☐ Intermittent	☐ Mild persistent	☐ Moderate persistent	☐ Severe persistent

Assessment form at different time points

Subject ID				
Group	☐ Intervention	☐ Control		
Time point<br>(Date: DD/MMM/YYYY)	☐ Baseline<br>(_ _/_ _ _/_ _ _ _)	☐ Day 60±7<br>(_ _/_ _ _/_ _ _ _)	☐ Day 120± 7<br>(_ _/_ _ _/_ _ _ _)	☐ Day 180±7<br>(_ _/_ _ _/_ _ _ _)
AR graded according to ARIA				
- Frequency ł	☐ Intermittent<br>☐ Persistent	☐ Intermittent<br>☐ Persistent	☐ Intermittent<br>☐ Persistent	☐ Intermittent<br>☐ Persistent
- Severity of AR§	☐ Mild<br>☐ Moderate-Severe	☐ Mild<br>☐ Moderate-Severe	☐ Mild<br>☐ Moderate-Severe	☐ Mild<br>☐ Moderate-Severe
Endoscopic assessment for Middle turbinate edema grading by physician	☐ Normal<br>☐ Focal<br>☐ Multifocal<br>☐ Diffuse<br>☐ Polypoid Edema.			☐ Normal<br>☐ Focal<br>☐ Multifocal<br>☐ Diffuse<br>☐ Polypoid Edema.
Family history of AR	☐ Yes<br>☐ No			
Self-reported triggering allergen (if known)	☐ Dust<br>☐ Food_________<br>☐ Air-cond. Room<br>☐ Domestic pet<br>☐ Drugs<br>___________			
Concomitant Medication of Interest*				
- Name of medication				
- Strength				
- Frequency				
- Dose				
- Route				
Complaints of medication* side effects experienced				

*Antihistamine, Corticosteroids, Anticholinergics, Leukotriene receptor antagonists, Nasal saline, Decongestants, Beta- blocker
ł Intermitent: < 4 days per week or < 4 weeks at a time; Persistent: ≥ 4 days per week, and ≥ 4 weeks at a time
§ Mild: Normal sleep, daily activities, work/school and no troublesome symptoms;
§ Moderate to severe: One or more of the symptoms including abnormal sleep, impairment of daily activities, sport, leisure, problem at work or school, troublesome symptoms.

<u>Assessment form at out-of-schedule visit at patient's nearest health instituition</u>

Subject ID				
Group	☐ Intervention	☐ Control		
Date of visit (DD/MMM/YYYY)	(_ _ / _ _ _/ _ _ _ _)	(_ _ / _ _ _/ _ _ _ _)	(_ _ / _ _ _/ _ _ _ _)	(_ _ / _ _ _/ _ _ _ _)
Health institution (name)				
Reason of visit				
AR graded according to ARIA				
- Frequency ł	☐ Intermittent<br>☐ Persistent	☐ Intermittent<br>☐ Persistent	☐ Intermittent<br>☐ Persistent	☐ Intermittent<br>☐ Persistent
- Severity of AR§	☐ Mild<br>☐ Moderate-Severe	☐ Mild<br>☐ Moderate-Severe	☐ Mild<br>☐ Moderate-Severe	☐ Mild<br>☐ Moderate-Severe
Medications prescribed				
- Name of medication				
- Strength				
- Frequency				
- Dose				
- Route				

Appendix E: Validated questionnaire assessing knowledge level

(English version)

We are interested to learn your respond for each statement.

For each statement, please circle "**Yes**", "**Not sure**" or "**No**" depending on your comprehension.

1) Nasal spray contains steroid	Yes	Not sure	No
2) Nasal steroid has long term side effects	Yes	Not sure	No
3) Nasal steroid is an effective treatment for allergic rhinitis	Yes	Not sure	No
4) I know the correct method of using the nasal steroid	Yes	Not sure	No

(Bahasa version)

Kami berminat untuk mengetahui maklum balas anda bagi setiap kenyataan.

Bagi setiap pernyataan di bawah, sila bulatkan "**Ya**", "**Tidak pasti**" atau "**Tidak**" bergantung kepada pemahaman anda.

1) Alat semburan hidung mengandungi steroid	Ya	Tidak Pasti	Tidak
2) Alat semburan hidung steroid mempunyai kesan sampingan jangka panjang	Ya	Tidak Pasti	Tidak
3) Alat semburan hidung steroid adalah rawatan yang berkesan untuk alahan hidung	Ya	Tidak Pasti	Tidak
4) Saya tahu kaedah yang betul menggunakan alat semburan hidung steroid	Ya	Tidak Pasti	Tidak

Appendix F: Total Nasal Symptom Score (TNSS) (English version)

TOTAL NASAL SYMPTOM SCORE

PLEASE ANSWER ALL QUESTIONS TO THE BEST OF YOUR ABILITY. This information will assist us in understanding and treating your symptoms.

1. Please rate how your **nasal congestion** has been over the past:	12 hours	Last 2 weeks
None	0 ○	0 ○
Mild (symptom clearly present but easily tolerated)	1 ○	1 ○
Moderate (symptom bothersome but tolerable)	2 ○	2 ○
Severe (symptom difficult to tolerate – interferes with activities)	3 ○	3 ○

2. Please rate how your **runny nose** has been over the past:	12 hours	Last 2 weeks
None	0 ○	0 ○
Mild (symptom clearly present but easily tolerated)	1 ○	1 ○
Moderate (symptom bothersome but tolerable)	2 ○	2 ○
Severe (symptom difficult to tolerate – interferes with activities)	3 ○	3 ○

3. Please rate how your **nasal itching** has been over the past:	12 hours	Last 2 weeks
None	0 ○	0 ○
Mild (symptom clearly present but easily tolerated)	1 ○	1 ○
Moderate (symptom bothersome but tolerable)	2 ○	2 ○
Severe (symptom difficult to tolerate – interferes with activities)	3 ○	3 ○

4. Please rate how your **sneezing** has been over the past:	12 hours	Last 2 weeks
None	0 ○	0 ○
Mild (symptom clearly present but easily tolerated)	1 ○	1 ○
Moderate (symptom bothersome but tolerable)	2 ○	2 ○
Severe (symptom difficult to tolerate – interferes with activities)	3 ○	3 ○

TOTAL SCORE: ____0________ / ____0_________

(Bahasa version)

JUMLAH SKOR SIMPTOM HIDUNG

SILA JAWAB SEMUA SOALAN DENGAN SEBAIK MUNGKIN. Informasi ini akan membantu kami memahami dan merawat simptom anda.

1. Sila nilaikan keadaan **hidung tersumbat** anda dalam tempoh:	12 jam lalu	2 minggu lalu
Tiada	0 ○	0 ○
Ringan (simptom jelas ada tetapi mudah diterima)	1 ○	1 ○
Sederhana (simptom mengganggu tetapi masih boleh diterima)	2 ○	2 ○
Teruk (simptom sukar diterima - mengganggu aktiviti)	3 ○	3 ○

2. Sila nilaikan keadaan **hidung berair** anda dalam tempoh:	12 jam lalu	2 minggu lalu
Tiada	0 ○	0 ○
Ringan (simptom jelas ada tetapi mudah diterima)	1 ○	1 ○
Sederhana (simptom mengganggu tetapi masih boleh diterima)	2 ○	2 ○
Teruk (simptom sukar diterima - mengganggu aktiviti)	3 ○	3 ○

3. Sila nilaikan **kegatalan hidung** anda dalam tempoh:	12 jam lalu	2 minggu lalu
Tiada	0 ○	0 ○
Ringan (simptom jelas ada tetapi mudah diterima)	1 ○	1 ○
Sederhana (simptom mengganggu tetapi masih boleh diterima)	2 ○	2 ○
Teruk (simptom sukar diterima - mengganggu aktiviti)	3 ○	3 ○

4. Sila nilaikan bagaimana **bersin** anda dalam tempoh:	12 jam lalu	2 minggu lalu
Tiada	0 ○	0 ○
Ringan (simptom jelas ada tetapi mudah diterima)	1 ○	1 ○
Sederhana (simptom mengganggu tetapi masih boleh diterima)	2 ○	2 ○
Teruk (simptom sukar diterima - mengganggu aktiviti)	3 ○	3 ○

JUMLAH MARKAH: ____0________ / ____0_________

Appendix G: Medication adherence

Nasal spray usage diary	
Subject ID:<br><br>Medication name:<br><br>Strength<br><br>Dosage:<br><br>Frequency:	*Instruction*<br><br>**Please check (√) on the box after each nasal spray use**

Week 1 [_ _/ _ _ to _ _/ _ _]	Mon	Tues	Wed	Thrus	Fri	Sat	Sun
Day time							
Night time							
Additional 2 Sprays							
Another 2 Additional Sprays							
Week 2 [_ _/ _ _ to _ _/ _ _]	Mon	Tues	Wed	Thrus	Fri	Sat	Sun
Day time							
Night time							
Additional 2 Sprays							
Another 2 Additional Sprays							
Week 3 [_ _/ _ _ to _ _/ _ _]	Mon	Tues	Wed	Thrus	Fri	Sat	Sun
Day time							
Night time							
Additional 2 Sprays							
Another 2 Additional Sprays							
Week 4 [_ _/ _ _ to _ _/ _ _]	Mon	Tues	Wed	Thrus	Fri	Sat	Sun
Day time							
Night time							
Additional 2 Sprays							
Another 2 Additional Sprays							

Nasal spray usage diary							
Subject ID:					*Instruction*<br>**Please check (√) on the box after each nasal spray use**		
Week 5<br>[_ _/ _ _ to _ _/ _ _]	Mon	Tues	Wed	Thrus	Fri	Sat	Sun
Day time							
Night time							
Additional 2 Sprays							
Another 2 Additional Sprays							
Week 6<br>[_ _/ _ _ to _ _/ _ _]	Mon	Tues	Wed	Thrus	Fri	Sat	Sun
Day time							
Night time							
Additional 2 Sprays							
Another 2 Additional Sprays							
Week 7<br>[_ _/ _ _ to _ _/ _ _]	Mon	Tues	Wed	Thrus	Fri	Sat	Sun
Day time							
Night time							
Additional 2 Sprays							
Another 2 Additional Sprays							
Week 8<br>[_ _/ _ _ to _ _/ _ _]	Mon	Tues	Wed	Thrus	Fri	Sat	Sun
Day time							
Night time							
Additional 2 Sprays							
Another 2 Additional Sprays							

Nasal spray usage diary							
Subject ID:					*Instruction*<br>**Please check (√) on the box after each nasal spray use**		
Week 9 [_ _/ _ _ to _ _/ _ _]	Mon	Tues	Wed	Thrus	Fri	Sat	Sun
Day time							
Night time							
Additional 2 Sprays							
Another 2 Additional Sprays							
Week 10 [_ _/ _ _ to _ _/ _ _]	Mon	Tues	Wed	Thrus	Fri	Sat	Sun
Day time							
Night time							
Additional 2 Sprays							
Another 2 Additional Sprays							
Week 11 [_ _/ _ _ to _ _/ _ _]	Mon	Tues	Wed	Thrus	Fri	Sat	Sun
Day time							
Night time							
Additional 2 Sprays							
Another 2 Additional Sprays							
Week 12 [_ _/ _ _ to _ _/ _ _]	Mon	Tues	Wed	Thrus	Fri	Sat	Sun
Day time							
Night time							
Additional 2 Sprays							
Another 2 Additional Sprays							

Nasal spray usage diary							
Subject ID:					*Instruction*<br>**Please check (√) on the box after each nasal spray use**		
Week 13<br>[_ _/ _ _ to _ _/ _ _]	Mon	Tues	Wed	Thrus	Fri	Sat	Sun
Day time							
Night time							
Additional 2 Sprays							
Another 2 Additional Sprays							
Week 14<br>[_ _/ _ _ to _ _/ _ _]	Mon	Tues	Wed	Thrus	Fri	Sat	Sun
Day time							
Night time							
Additional 2 Sprays							
Another 2 Additional Sprays							
Week 15<br>[_ _/ _ _ to _ _/ _ _]	Mon	Tues	Wed	Thrus	Fri	Sat	Sun
Day time							
Night time							
Additional 2 Sprays							
Another 2 Additional Sprays							
Week 16<br>[_ _/ _ _ to _ _/ _ _]	Mon	Tues	Wed	Thrus	Fri	Sat	Sun
Day time							
Night time							
Additional 2 Sprays							
Another 2 Additional Sprays							

Nasal spray usage diary							
Subject ID:					*Instruction*<br>**Please check (√) on the box after each nasal spray use**		
Week 17<br>[_ _/ _ _ to _ _/ _ _]	Mon	Tues	Wed	Thrus	Fri	Sat	Sun
Day time							
Night time							
Additional 2 Sprays							
Another 2 Additional Sprays							
Week 18<br>[_ _/ _ _ to _ _/ _ _]	Mon	Tues	Wed	Thrus	Fri	Sat	Sun
Day time							
Night time							
Additional 2 Sprays							
Another 2 Additional Sprays							
Week 19<br>[_ _/ _ _ to _ _/ _ _]	Mon	Tues	Wed	Thrus	Fri	Sat	Sun
Day time							
Night time							
Additional 2 Sprays							
Another 2 Additional Sprays							
Week 20<br>[_ _/ _ _ to _ _/ _ _]	Mon	Tues	Wed	Thrus	Fri	Sat	Sun
Day time							
Night time							
Additional 2 Sprays							
Another 2 Additional Sprays							

Nasal spray usage diary							
Subject ID:					*Instruction*<br>**Please check (√) on the box after each nasal spray use**		
Week 21<br>[_ _/ _ _ to _ _/ _ _]	Mon	Tues	Wed	Thrus	Fri	Sat	Sun
Day time							
Night time							
Additional 2 Sprays							
Another 2 Additional Sprays							
Week 22<br>[_ _/ _ _ to _ _/ _ _]	Mon	Tues	Wed	Thrus	Fri	Sat	Sun
Day time							
Night time							
Additional 2 Sprays							
Another 2 Additional Sprays							
Week 23<br>[_ _/ _ _ to _ _/ _ _]	Mon	Tues	Wed	Thrus	Fri	Sat	Sun
Day time							
Night time							
Additional 2 Sprays							
Another 2 Additional Sprays							
Week 24<br>[_ _/ _ _ to _ _/ _ _]	Mon	Tues	Wed	Thrus	Fri	Sat	Sun
Day time							
Night time							
Additional 2 Sprays							
Another 2 Additional Sprays							

(Bahasa version) Kepatuhan pengambilan ubat

Diari penggunaan alat semburan hidung							
Subjek ID:<br>Nama Ubat:<br>Kekuatan<br>Dos:<br>Kekerapan:					*Arahan*<br>**Sila tandakan (√) kotak selepas setiap penggunaan alat semburan hidung**		
Minggu 1<br>[_ _/ _ _ hingga _ _/ _ _]	Isnin	Selasa	Rabu	Khamis	Jumaat	Sabtu	Ahad
Waktu siang							
Waktu malam							
2 semburan tambahan							
Lagi 2 Semburan Tambahan							
Minggu 2<br>[_ _/ _ _ hingga _ _/ _ _]	Isnin	Selasa	Rabu	Khamis	Jumaat	Sabtu	Ahad
Waktu siang							
Waktu malam							
2 semburan tambahan							
Lagi 2 Semburan Tambahan							
Minggu 3<br>[_ _/ _ _ hingga _ _/ _ _]	Isnin	Selasa	Rabu	Khamis	Jumaat	Sabtu	Ahad
Waktu siang							
Waktu malam							
2 semburan tambahan							
Lagi 2 Semburan Tambahan							
Minggu 4<br>[_ _/ _ _ hingga _ _/ _ _]	Isnin	Selasa	Rabu	Khamis	Jumaat	Sabtu	Ahad
Waktu siang							
Waktu malam							
2 semburan tambahan							
Lagi 2 Semburan Tambahan							

Diari penggunaan alat semburan hidung							
Subjek ID:					*Arahan*<br>**Sila tandakan (√) kotak selepas setiap penggunaan alat semburan hidung**		
Minggu 5<br>[_ _/ _ _ hingga _ _/ _ _]	Isnin	Selasa	Rabu	Khamis	Jumaat	Sabtu	Ahad
Waktu siang							
Waktu malam							
2 semburan tambahan							
Lagi 2 Semburan Tambahan							
Minggu 6<br>[_ _/ _ _ hingga _ _/ _ _]	Isnin	Selasa	Rabu	Khamis	Jumaat	Sabtu	Ahad
Waktu siang							
Waktu malam							
2 semburan tambahan							
Lagi 2 Semburan Tambahan							
Minggu 7<br>[_ _/ _ _ hingga _ _/ _ _]	Isnin	Selasa	Rabu	Khamis	Jumaat	Sabtu	Ahad
Waktu siang							
Waktu malam							
2 semburan tambahan							
Lagi 2 Semburan Tambahan							
Minggu 8<br>[_ _/ _ _ hingga _ _/ _ _]	Isnin	Selasa	Rabu	Khamis	Jumaat	Sabtu	Ahad
Waktu siang							
Waktu malam							
2 semburan tambahan							
Lagi 2 Semburan Tambahan							

Diari penggunaan alat semburan hidung							
Subjek ID:					*Arahan*<br>**Sila tandakan (√) kotak selepas setiap penggunaan alat semburan hidung**		
Minggu 9<br>[_ _/ _ _ hingga _ _/ _ _]	Isnin	Selasa	Rabu	Khamis	Jumaat	Sabtu	Ahad
Waktu siang							
Waktu malam							
2 semburan tambahan							
Lagi 2 Semburan Tambahan							
Minggu 10<br>[_ _/ _ _ hingga _ _/ _ _]	Isnin	Selasa	Rabu	Khamis	Jumaat	Sabtu	Ahad
Waktu siang							
Waktu malam							
2 semburan tambahan							
Lagi 2 Semburan Tambahan							
Minggu 11<br>[_ _/ _ _ hingga _ _/ _ _]	Isnin	Selasa	Rabu	Khamis	Jumaat	Sabtu	Ahad
Waktu siang							
Waktu malam							
2 semburan tambahan							
Lagi 2 Semburan Tambahan							
Minggu 12<br>[_ _/ _ _ hingga _ _/ _ _]	Isnin	Selasa	Rabu	Khamis	Jumaat	Sabtu	Ahad
Waktu siang							
Waktu malam							
2 semburan tambahan							
Lagi 2 Semburan Tambahan							

Diari penggunaan alat semburan hidung							
Subjek ID:					*Arahan*<br>**Sila tandakan (√) kotak selepas setiap penggunaan alat semburan hidung**		
Minggu 13<br>[_ _/ _ _ hingga _ _/ _ _]	Isnin	Selasa	Rabu	Khamis	Jumaat	Sabtu	Ahad
Waktu siang							
Waktu malam							
2 semburan tambahan							
Lagi 2 Semburan Tambahan							
Minggu 14<br>[_ _/ _ _ hingga _ _/ _ _]	Isnin	Selasa	Rabu	Khamis	Jumaat	Sabtu	Ahad
Waktu siang							
Waktu malam							
2 semburan tambahan							
Lagi 2 Semburan Tambahan							
Minggu 15<br>[_ _/ _ _ hingga _ _/ _ _]	Isnin	Selasa	Rabu	Khamis	Jumaat	Sabtu	Ahad
Waktu siang							
Waktu malam							
2 semburan tambahan							
Lagi 2 Semburan Tambahan							
Minggu 16<br>[_ _/ _ _ hingga _ _/ _ _]	Isnin	Selasa	Rabu	Khamis	Jumaat	Sabtu	Ahad
Waktu siang							
Waktu malam							
2 semburan tambahan							
Lagi 2 Semburan Tambahan							

Diari penggunaan alat semburan hidung							
Subjek ID:					*Arahan*<br>**Sila tandakan (√) kotak selepas setiap penggunaan alat semburan hidung**		
Minggu 17<br>[_ _/ _ _ hingga _ _/ _ _]	Isnin	Selasa	Rabu	Khamis	Jumaat	Sabtu	Ahad
Waktu siang							
Waktu malam							
2 semburan tambahan							
Lagi 2 Semburan Tambahan							
Minggu 18<br>[_ _/ _ _ hingga _ _/ _ _]	Isnin	Selasa	Rabu	Khamis	Jumaat	Sabtu	Ahad
Waktu siang							
Waktu malam							
2 semburan tambahan							
Lagi 2 Semburan Tambahan							
Minggu 19<br>[_ _/ _ _ hingga _ _/ _ _]	Isnin	Selasa	Rabu	Khamis	Jumaat	Sabtu	Ahad
Waktu siang							
Waktu malam							
2 semburan tambahan							
Lagi 2 Semburan Tambahan							
Minggu 20<br>[_ _/ _ _ hingga _ _/ _ _]	Isnin	Selasa	Rabu	Khamis	Jumaat	Sabtu	Ahad
Waktu siang							
Waktu malam							
2 semburan tambahan							
Lagi 2 Semburan Tambahan							

1717

Diari penggunaan alat semburan hidung							
Subjek ID:					*Arahan*<br>**Sila tandakan (√) kotak selepas setiap penggunaan alat semburan hidung**		
Minggu 21<br>[_ _/ _ _ hingga _ _/ _ _]	Isnin	Selasa	Rabu	Khamis	Jumaat	Sabtu	Ahad
Waktu siang							
Waktu malam							
2 semburan tambahan							
Lagi 2 Semburan Tambahan							
Minggu 22<br>[_ _/ _ _ hingga _ _/ _ _]	Isnin	Selasa	Rabu	Khamis	Jumaat	Sabtu	Ahad
Waktu siang							
Waktu malam							
2 semburan tambahan							
Lagi 2 Semburan Tambahan							
Minggu 23<br>[_ _/ _ _ hingga _ _/ _ _]	Isnin	Selasa	Rabu	Khamis	Jumaat	Sabtu	Ahad
Waktu siang							
Waktu malam							
2 semburan tambahan							
Lagi 2 Semburan Tambahan							
Minggu 24<br>[_ _/ _ _ hingga _ _/ _ _]	Isnin	Selasa	Rabu	Khamis	Jumaat	Sabtu	Ahad
Waktu siang							
Waktu malam							
2 semburan tambahan							
Lagi 2 Semburan Tambahan							

1718

Appendix H: Health Questionnaire (EQ-5D-5L)

Health Questionnaire

English version for Malaysia

Malaysia (English) © 2010 EuroQol Group EQ-5D™ is a trade mark of the EuroQol Group

Under each heading, please tick the ONE box that best describes your health TODAY.

MOBILITY

I have no problems in walking about ❑

I have slight problems in walking about ❑

I have moderate problems in walking about ❑

I have severe problems in walking about ❑

I am unable to walk about ❑

SELF-CARE

I have no problems cleaning my body or dressing myself ❑

I have slight problems cleaning my body or dressing myself ❑

I have moderate problems cleaning my body or dressing myself ❑

I have severe problems cleaning my body or dressing myself ❑

I am unable to clean my body or dress myself ❑

USUAL ACTIVITIES *(e.g. work, study, housework, family or leisure activities)*

I have no problems doing my usual activities ❑

I have slight problems doing my usual activities ❑

I have moderate problems doing my usual activities ❑

I have severe problems doing my usual activities ❑

I am unable to do my usual activities ❑

PAIN / DISCOMFORT

I have no pain or discomfort ❑

I have slight pain or discomfort ❑

I have moderate pain or discomfort ❑

I have severe pain or discomfort ❑

I have extreme pain or discomfort ❑

ANXIETY / DEPRESSION

I am not anxious or depressed ❑

I am slightly anxious or depressed ❑

I am moderately anxious or depressed ❑

I am severely anxious or depressed ❑

I am extremely anxious or depressed ❑

Malaysia (English) © 2010 EuroQol Group EQ-5D™ is a trade mark of the EuroQol Group

- We would like to know how good or bad your health is TODAY.
- This scale is numbered from 0 to 100.
- 100 means the best health you can imagine.
  0 means the worst health you can imagine.
- Mark an X on the scale to indicate how your health is TODAY.
- Now, please write the number you marked on the scale in the box below.

Malaysia (English) © 2010 EuroQol Group EQ-5D™ is a trade mark of the EuroQol Group

Soal Selidik Kesihatan

Versi Bahasa Melayu untuk Malaysia

(Malay version for Malaysia)

Di bawah setiap tajuk, sila tandakan SATU kotak yang menggambarkan keadaan kesihatan anda HARI INI dengan paling tepat.

PERGERAKAN

Saya tidak menghadapi masalah untuk berjalan ❑
Saya menghadapi sedikit masalah untuk berjalan ❑
Saya menghadapi masalah yang sederhana untuk berjalan ❑
Saya menghadapi masalah yang teruk untuk berjalan ❑
Saya tidak berupaya untuk berjalan ❑

PENJAGAAN DIRI

Saya tidak menghadapi masalah untuk membersihkan diri atau memakai sendiri pakaian saya ❑
Saya menghadapi sedikit masalah untuk membersihkan diri atau memakai sendiri pakaian saya ❑
Saya menghadapi masalah yang sederhana untuk membersihkan diri atau memakai sendiri pakaian saya ❑
Saya menghadapi masalah yang teruk untuk membersihkan diri atau memakai sendiri pakaian saya ❑
Saya tidak berupaya untuk membersihkan diri atau memakai sendiri pakaian saya ❑

AKTIVITI-AKTIVITI BIASA *(misalnya bekerja, belajar, membuat kerja rumah, aktiviti-aktiviti keluarga atau masa lapang)*

Saya tidak menghadapi masalah untuk melakukan aktiviti-aktiviti biasa saya ❑
Saya menghadapi sedikit masalah untuk melakukan aktiviti-aktiviti biasa saya ❑
Saya menghadapi masalah yang sederhana untuk melakukan aktiviti-aktiviti biasa saya ❑
Saya menghadapi masalah yang teruk untuk melakukan aktiviti-aktiviti biasa saya ❑
Saya tidak berupaya untuk melakukan aktiviti-aktiviti biasa saya ❑

KESAKITAN / KETIDAKSELESAAN

Saya tidak berasa sakit atau tidak selesa ❑
Saya berasa sakit atau tidak selesa sedikit ❑
Saya berasa sakit atau tidak selesa yang sederhana ❑
Saya berasa sakit atau tidak selesa yang teruk ❑
Saya berasa sakit atau tidak selesa yang teramat sangat ❑

KERISAUAN / KEMURUNGAN

Saya tidak berasa risau atau murung ❑
Saya berasa risau atau murung sedikit ❑
Saya berasa risau atau murung yang sederhana ❑
Saya berasa risau atau murung yang teruk ❑
Saya berasa risau atau murung yang teramat sangat ❑

- Kami ingin tahu betapa baik atau tidak baik kesihatan anda pada HARI INI.
- Skala ini bernombor dari 0 hingga 100.
- 100 bermaksud kesihatan paling baik yang boleh anda bayangkan.
  0 bermaksud kesihatan paling teruk yang boleh anda bayangkan.
- Tandakan X pada skala untuk menandakan bagaimana kesihatan anda pada HARI INI.
- Sekarang, sila tuliskan nombor yang anda tandakan pada skala dalam kotak di bawah.

Appendix I: Checklist of implementation of pharmacist-led education intervention

Subject ID: _____	Please tick when done			
	_ _/_ _	_ _/_ _	_ _/_ _	_ _/_ _
Items	Day 0	Day 60±7	Day 120±7,	Day 180±7
1. Show the patient education to participants				
2. Assess the nasal steroid technique				
3. Assess patient's nasal spray adherence				
4. If non-adherent, assess patients' concern to medication. Circle N/A is patient adhered to nasal spray		N/A	N/A	N/A
a. To discuss with the doctor if uncertain about addressing patients' concern		N/A	N/A	N/A
b. To explain the importance of adherence		N/A	N/A	N/A
5. Emphasize the importance of allergen identification and avoidance				
6. Encourage patient to visit the patient education materials from time to time. Assess if they got question about the education material				
7. If patient has co-existing of asthma and allergic rhinitis, emphasize the importance to adhere to inhaler.	N/A	N/A	N/A	` N/A
a. If asthma exacerbations occur, advise the patient to go to the emergency room immediately	N/A	N/A	N/A	N/A
