# Supplementary material for: Pharmacist-Led Education Intervention for Adults With Allergic Rhinitis: A Randomized Clinical Trial
Source: JAMA Netw Open. 2025 Jul 16;8(7):e2517160. doi: 10.1001/jamanetworkopen.2025.17160 (PMC12268493; doi:10.1001/jamanetworkopen.2025.17160)
Supplement: Supplement 2. — eTable 1. Estimates of Prior Distributions for Each Primary End Point eTable 2. Primary End Points for Efficacy Evaluation eTable 3. Participants’ Primary Outcomes (PP vs ITT) eTable 4. Demographic Characteristics of Study Dropouts Versus Completers eTable 5. Safety [file jamanetwopen-e2517160-s002.pdf]

Supplementary Online Content

Chew CC, Lim XJ, Letchumanan P, Rajan P, Chong CP. Pharmacist-led education intervention for adults with allergic rhinitis: a randomized clinical trial. *JAMA Netw Open.* 2025;8(7):e2517160. doi:10.1001/jamanetworkopen.2025.17160

eTable 1. Estimates of Prior Distributions for Each Primary End Point

eTable 2. Primary End Points for Efficacy Evaluation

eTable 3. Participants' Primary Outcomes (PP vs ITT)

eTable 4. Demographic Characteristics of Study Dropouts Versus Completers

eTable 5. Safety

This supplementary material has been provided by the authors to give readers additional information about their work.

© 2025 Chew CC et al. *JAMA Network Open.*

eTable 1. Estimates of Prior Distributions for Each Primary End Point

	ITT	
**Primary endpoints**	***Estimate (SE) [95% CrI]***	**Prior Parameters**
		Weakly informative prior: Fixed effect normal (0, 1), Random effects SD Exp (1); Non-informative prior: Fixed effect normal (0, 100),
**Knowledge**		
Weakly informative	-0.02 (0.03) [-0.09 to 0.04]	Intercept N (0, 1), Residual SD Exp (1)
Non-informative	-0.02 (0.03) [-0.09 to 0.04]	Intercept N (0, 10), Random effects SD HC (0, 5), residual SD Exp (0.1).
**TNSS at past 12 hours**		
Weakly informative	0.08 (0.05) [-0.03 to 0.18]	Intercept N (1, 2), Zero-inflation Beta (2, 2), Shape Gamma (2, 0.1)
Non-informative	0.08 (0.05) [-0.03 to 0.18]	Intercept N (0, 10), Zero-inflation Beta (1,1), Random effects SD Exp (0.001), Shape Exp (0.001)
**TNSS at past 2 weeks**		
Weakly informative	0.14 (0.06) [0.03 to 0.25]	Intercept N (1, 2), Zero-inflation Beta (1,3), Shape Gamma (2,0.1)
Non-informative	0.14 (0.06) [0.03 to 0.25]	Intercept N (0, 10), zero-inflation Beta (1, 1), Random effects SD HC (0, 5), Shape Gamma (0.01, 0.01).
**Medication adherence**		
Weakly informative	0.15 (0.12) [-0.08 to 0.38]	Intercept N (2,1), Zero-inflation Beta (2,3), Shape Gamma (3,0.1)
Non-informative	0.15 (0.12) [-0.08 to 0.38]	intercept N (0,10), zero-inflation Beta (1,1), Random effects SD Exp (0.001), Shape Exp (0.001)
**QoL, EQ-5D utility scores**		
Weakly informative	0.00 (0.01) [-0.02 to 0.02]	Intercept N (0.7,0.3), Residual SD Exp (1), Skewness N (0,1)
Non-informative	0.00 (0.01) [-0.01 to 0.01]	Intercept N (0, 10), Random/residual SD HC (0, 5), Skewness N (0, 10).
**QoL, EQ-VAS**		
Weakly informative	0.07 (0.04) [-0.01 to 0.14]	Intercept N (4.2, 0.5), Zero-inflation Beta (2, 10), Shape Gamma (3, 0.1).
Non-informative	0.07 (0.04) [-0.01 to 0.14]	Intercept N (0, 10), Zero-inflation Beta (1, 1), Random effects SD HC (0, 5), Shape Gamma (0.01, 0.01).

Non-informative prior was chosen because of the absence of prior information, given that the model was newly developed. Estimates based on the non-informative prior were consistent with those based on the weakly informative prior. Abbreviation. ITT: Intention-to-treat, SD: Standard deviation, N: Normal, Exp: Exponential, HC = Half-Cauchy, SE: standard error, Estimate: Regression coefficient estimate, CrI: Credible interval

© 2025 Chew CC et al. *JAMA Network Open.*

eTable 2. Primary End Points for Efficacy Evaluation

**Primary endpoints**	**ITT**	**PP**
	Estimate (SE) [95% CrI]	***Estimate (SE) [95% CrI]***
**Knowledge level**		
Intercept	1.78 (0.02) [1.73 to 1.82]	1.77 (0.02) [1.73 to 1.82]
Intervention group	-0.02 (0.03) [-0.09 to 0.04]	-0.02 (0.03) [-0.09 to 0.05]
Day 120±7	0.07 (0.03) [0.02 to 0.12]	0.08 (0.03) [0.03 to 0.13]
Day 180±7	0.08 (0.03) [0.03 to 0.13]	0.08 (0.03) [0.03 to 0.13]
**TNSS at past 12 hours**		
Intercept	2.09 (0.04) [2.02 to 2.17]	2.09 (0.04) [2.01 to 2.17]
Intervention group	0.08 (0.05) [-0.03 to 0.18]	0.07 (0.05) [-0.03 to 0.18]
Day 60±7	0.15 (0.05) [0.06 to 0.25]	0.15 (0.05) [0.05 to 0.25]
Day 120±7	0.18 (0.05) [0.09 to 0.28]	0.19 (0.05) [0.09 to 0.28]
Day 180±7	0.13 (0.05) [0.03 to 0.22]	0.11 (0.05) [0.01 to 0.21]
**TNSS at past 2 weeks**		
Intercept	1.94 (0.04) [1.86 to 2.02]	1.94 (0.04) [1.85 to 2.02]
Intervention group	0.14 (0.06) [0.03 to 0.25]	0.13 (0.06) [0.02 to 0.25]
Day 60±7	0.18 (0.05) [0.08 to 0.28]	0.18 (0.05) [0.08 to 0.28]
Day 120±7	0.21 (0.05) [0.11 to 0.31]	0.21 (0.05) [0.11 to 0.31]
Day 180±7	0.11 (0.05) [0.01 to 0.21]	0.10 (0.05) [-0.01 to 0.20]
**Medication adherence**		
Intercept	3.60 (0.08) [3.44 to 3.76]	3.59 (0.08 [3.42 to 3.75]
Intervention group	0.15 (0.12) [-0.08 to 0.38]	0.16 (0.12) [-0.08 to 0.39]
Day 120±7	0.62 (0.03) [0.57 to 0.68]	0.64 (0.03) [0.58 to 0.69]
Day 180±7	1.08 (0.03) [1.02 to 1.13]	1.08 (0.03) [1.02 to 1.14]
**QoL, EQ-5D utility scores**		
Intercept	0.89 (0.00) [0.88 to 0.90]	0.89 (0.01) [0.88 to 0.90]
Intervention group	0.00 (0.01) [-0.01 to 0.01]	0.00 (0.01) [-0.01 to 0.01]
Day 60±7	0.00 (0.01) [-0.01 to 0.01]	0.00 (0.01) [-0.01 to 0.01]
Day 120±7	0.00 (0.01) [-0.01 to 0.01]	0.00 (0.01) [-0.01 to 0.01]
Day 180±7	0.00 (0.01) [-0.01 to 0.01]	0.00 (0.01) [-0.01 to 0.01]
**QoL, EQ-VAS**		
Intercept	4.31 (0.03) [4.25 to 4.36]	4.30 (0.03) [4.25 to 4.36]
Intervention group	0.07 (0.04) [-0.01 to 0.14]	0.07 (0.04) [-0.02 to 0.15]
Day 60±7	0.08 (0.03) [0.01 to 0.14]	0.08 (0.03) [0.01 to 0.15]
Day 120±7	0.08 (0.03) [0.01 to 0.14]	0.08 (0.03) [0.01 to 0.14]
Day 180±7	0.09 (0.03) [0.03 to 0.15]	0.09 (0.03) [0.03 to 0.16]

Analyzed using Bayesian Generalized Linear Mixed Models with the control group as the reference, baseline was used as the reference for the dependent variable. For the analysis of medication adherence, Day 60±7 was set as the reference for the dependent variable. ITT: Intention-to-treat analysis, PP: Per-protocol analysis. SE: standard error, Estimate: Regression coefficient estimate, CrI: Credible interval, TNSS: Total nasal symptoms score; EQ-5D: Euro QoL 5-Dimensions, 5-Levels questionnaire, EQ-VAS: EQ-visual analogue scales.

© 2025 Chew CC et al. *JAMA Network Open.*

eTable 3. Participants' Primary Outcomes (PP vs ITT)

Primary endpoints	Day 60±7 median (IQR)			Day 120±7 median (IQR)			Day 180±7 median (IQR)		
	Intervention	Control	P-value	Intervention	Control	P-value	Intervention	Control	P-value
**Population, n (%)**									
PP	77 (50.7)	75 (49.3)	0.908[a]	76 (51.0)	73 (49.0)	0.853[a]	76 (49.7)	77 (50.3)	0.853[a]
ITT	77 (50.0)	77 (50.0)		77 (50.0)	77 (50.0)		77 (50.0)	77 (50.0)	
**Knowledge level**									
PP	-	-		7.00 (1.00)	6.00 (1.00)	0.965[b]	7.00 (2.00)	6.00 (1.00)	0.970[b]
ITT	-	-		7.00 (1.00)	6.00 (1.00)		7.00 (2.00)	6.00 (1.00)	
**TNSS at past 12 hours**									
PP	10.00 (3.00)	10.00 (3.00)	0.997[b]	11.00 (3.00)	11.00 (2.00)	0.996[b]	11.00 (2.75)	10.00 (3.50)	0.713[b]
ITT	10.00 (3.00)	10.00 (3.00)		11.00 (3.00)	11.00 (2.00)		11.00 (2.50)	10.00 (4.00)	
**TNSS at past 2 weeks**									
PP	10.00 (3.00)	9.00 (2.50)	0.934[b]	10.00 (3.00)	9.00 (4.00)	0.921[b]	10.00 (3.00)	8.00 (4.50)	0.906[b]
ITT	10.00 (3.00)	9.00 (2.50)		10.00 (3.00)	9.00 (4.00)		10.00 (3.00)	8.00 (4.00)	
**Medication adherence**									
PP	54.0 (26.0)	50.0 (35.0)	0.842 [b]	110.5 (54.25)	105.0 (75.0)	0.997[b]	162.5 (85.75)	154.0 (110.5)	0.973[b]
ITT	54.0 (26.0)	50.0 (35.5)		111.0 (54.0)	101.0 (75.0)		164.0 (84.50)	154.0 (110.5)	
**QoL, EQ-5D utility scores**									
PP	1.00 (0.00)	1.00 (0.08)	0.873[b]	1.00 (0.08)	1.00 (0.09)	0.904[b]	1.00 (0.08)	1.00 (0.08)	0.965[b]
ITT	1.00 (0.00)	1.00 (0.08)		1.00 (0.08)	1.00 (0.08)		1.00 (0.08)	1.00 (0.08)	
**QoL, EQ-VAS**									
PP	90.00 (20.00)	80.00 (15.00)	0.860[b]	90.00 (15.00)	80.00 (20.00)	0.998[b]	90.00 (15.00)	85.00 (12.50)	0.933[b]
ITT	90.00 (20.00)	80.00 (12.50)		90.00 (15.00)	80.00 (20.00)		90.00 (16.50)	85.00 (12.50)	

TNSS: Total nasal symptoms score, QoL: Quality of Life, EQ-5D: Euro QoL 5-Dimensions, 5-Levels questionnaire, EQ-VAS: EQ-visual analogue scales. ITT: Intention-to-treat analysis, PP: Per-Protocol analysis, IQR: Interquartile range, [a]Chi-square test, [b]Mann-Whitney U-test, ITT Imputation was performed by Last Observation Carried Forward (LOCF).

© 2025 Chew CC et al. *JAMA Network Open.*

eTable 4. Demographic Characteristics of Study Dropouts Versus Completers

Variables	Lost to follow-up in any time points<br>n=5, n (%)	Completed the procedures<br>n=149, n (%)	*p*-value
Age (median, IQR)	42.00, 36.00	44.00, 28.00	0.156[a]
Gender (female)	3 (60.0)	94 (63.1)	1.000[c]
Ethnicity			
Malay	4 (80.0)	77 (51.7)	
Chinese	1 (20.0)	43 (28.9)	0.608[c]
Indian	0 (0.0)	27 (18.1)	
Others	0 (0.0)	2 (1.3)	
Marital status			
Single	2 (40.0)	39 (26.2)	0.637[c]
Married	3 (60.0)	108 (72.5)	
Divorced	0 (0.0)	2 (1.3)	
Highest Educational level			
Primary	0 (0.0)	12 (8.1)	0.779[c]
Secondary	3 (60.0)	64 (43.0)	
Tertiary	2 (40.0)	73 (49.0)	
Occupation			0.439[c]
Managerial	2 (40.0)	40 (26.8)	
Intermediate and small employer	1 (20.0)	17 (11.4)	
Lower supervisory, technical, Semi- routine or routine	1 (20.0)	21 (14.1)	
Unemployed, retired or student	1 (20.0)	71 (47.7)	
Work hour	N=4	N=78	
< 45 hours per week	4 (100.0)	56 (71.8)	0.299[c]
⩾ 45 hours per week	0 (0.0)	22 (28.2)	
Smoking status			
Never smoked	5 (100.0)	135 (90.6)	1.000[c]
Current smoker	0 (0.0)	7 (4.7)	
Former smoker	0 (0.0)	7 (4.7)	
Pets at home	2 (40.0)	50 (33.5)	1.000[c]
Food allergy	3 (60.0)	77 (51.6)	1.000[c]
Allergy to other allergens	4 (80.0)	109 (73.1)	1.000[c]
History of nasal surgery	5 (100.0)	12 (8.1)	1.000[c]
Family history of allergy	2 (40.0)	67 (45.0)	1.000[c]
Comorbid of asthma	1 (20.0)	29 (19.5)	1.000[c]
INCS prescribed at baseline			1.000[c]
Mometasone Furoate 50mcg	5 (100.0)	122 (81.9)	

© 2025 Chew CC et al. *JAMA Network Open.*

Variables	Lost to follow-up in any time points<br>n=5, n (%)	Completed the procedures<br>n=149, n (%)	*p*-value
Fluticasone Furoate 27.5mcg	0 (0.0)	24 (16.1)	
Budesonide 64 mcg	0 (0.0)	3 (2.0)	
Oral Antihistamine			
Loratadine 10 mg	3 (60.0)	120 (80.5)	0.264[c]
Others (e.g. Levocetrizine)	2 (40.0)	29 (19.5)	

Age was reported as median (IQR) due to only 5 participants in the group with missing data, and the group without missing data showed significant deviation from normality (p = 0.012), [a]Mann-Whitney U-test, [b]Chi-square test, [c]Fisher exact test.

© 2025 Chew CC et al. *JAMA Network Open.*

eTable 5. Safety

Variables	Count, n (%)	Exp (B)	95% Wald CI for Exp (B)	*p*-value[a]
Out-of-scheduled health facilities visit				
Intervention	24 (40.7)	1		
Control	35 (59.3)	1.46	0.87, 2.45	0.155
Number of other medications intake				
Intervention	50 (46.7)	1		
Control	57 (53.3)	1.14	0.78, 1.67	0.499
Number of traditional and complementary product / procedures				
Intervention	11 (33.3)	1		
Control	22 (66.7)	2.00	0.97, 4.12	0.061
Number of INCS side effects				
Intervention	27 (35.5)	1		
Control	49 (64.5)	1.82	1.14, 2.90	0.013[b]
Number of oral antihistamine side effects				
Intervention	15 (40.5)	1		
Control	22 (59.5)	1.47	0.76, 2.83	0.253

[a]Analyzed with Poisson regression analysis. [b]Statistically significant differences between the two groups. INCS: Intranasal corticosteroid, Exp (B): Exponentiated Regression Coefficient, 95% Wald CI: 95% Wald Confidence Interval.

© 2025 Chew CC et al. *JAMA Network Open.*
