# Supplementary material for: Pharmacist-Led Education Intervention for Adults With Allergic Rhinitis: A Randomized Clinical Trial
Source: JAMA Netw Open. 2025 Jul 16;8(7):e2517160. doi: 10.1001/jamanetworkopen.2025.17160 (PMC12268493; doi:10.1001/jamanetworkopen.2025.17160)
Supplement: Supplement 3. — Data Sharing Statement [file jamanetwopen-e2517160-s003.pdf]

Data Sharing Statement

Chew. Pharmacist-Led Education Intervention for Adults With Allergic Rhinitis. *JAMA Netw Open*. Published July 16, 2025. doi:10.1001/jamanetworkopen.2025.17160

Data
Additional Information: ClinicalTrials.gov Identifier: NCT06027736, URL: https://clinicaltrials.gov/study/NCT06027736
Data available: No

Additional Information
Explanation for why data not available: The data belongs to the Clinical Research Centre under the Malaysian Ministry of Health. This research has been approved by the Medical Research and Ethics Committee, Ministry of Health Malaysia, which specifies that all records and data are to be kept strictly confidential and can only be used for the purpose of this study, and all precautions are to be taken to maintain data confidentiality. There, to ensure the data collected is kept confidential, it cannot be shared publicly. However, with a reasonable request, should any party require the data, they can send their request to Sector for Biostatistics & Data Repository, Office of NIH Manager, National Institutes of Health, Ministry of Health Malaysia, and with the permission from the Malaysian Director General of Health prior to its being shared with any party.
